# A rapid advice guideline for the diagnosis and treatment of 2019 novel coronavirus (2019-nCoV) infected pneumonia (standard version)

**DOI:** 10.1186/s40779-020-0233-6

**Published:** 2020-02-06

**Authors:** Ying-Hui Jin, Lin Cai, Zhen-Shun Cheng, Hong Cheng, Tong Deng, Yi-Pin Fan, Cheng Fang, Di Huang, Lu-Qi Huang, Qiao Huang, Yong Han, Bo Hu, Fen Hu, Bing-Hui Li, Yi-Rong Li, Ke Liang, Li-Kai Lin, Li-Sha Luo, Jing Ma, Lin-Lu Ma, Zhi-Yong Peng, Yun-Bao Pan, Zhen-Yu Pan, Xue-Qun Ren, Hui-Min Sun, Ying Wang, Yun-Yun Wang, Hong Weng, Chao-Jie Wei, Dong-Fang Wu, Jian Xia, Yong Xiong, Hai-Bo Xu, Xiao-Mei Yao, Yu-Feng Yuan, Tai-Sheng Ye, Xiao-Chun Zhang, Ying-Wen Zhang, Yin-Gao Zhang, Hua-Min Zhang, Yan Zhao, Ming-Juan Zhao, Hao Zi, Xian-Tao Zeng, Yong-Yan Wang, Xing-Huan Wang, , for the Zhongnan Hospital of Wuhan University Novel Coronavirus Management and Research Team, Evidence-Based Medicine Chapter of China International Exchange and Promotive Association for Medical and Health Care (CPAM)

**Affiliations:** 1 Center for Evidence-Based and Translational Medicine, Zhongnan Hospital of Wuhan University, Wuhan, 430071 China; 2 Institute of Hospital Management, Wuhan University, Wuhan, 430071 China; 3 Department of Respiratory Medicine, Zhongnan Hospital of Wuhan University, Wuhan, 430071 China; 4 Department of Pharmacy, Zhongnan Hospital of Wuhan University, Wuhan, 430071 China; 5 Institute of Evidence-Based Medicine and Knowledge Translation, Henan University, Kaifeng, 475000 China; 6 China Academy of Chinese Medical Sciences, Beijing, 100700 China; 7 China Center for Evidence Based Traditional Chinese Medicine (CCEBTCM), Beijing, 100700 China; 8 Department of Critical Care Medicine, Zhongnan Hospital of Wuhan University, Wuhan, 430071 China; 9 Department of Clinical Laboratory, Zhongnan Hospital of Wuhan University, Wuhan, 430071 China; 10 Department of Infectious Diseases, Zhongnan Hospital of Wuhan University, Wuhan, 430071 China; 11 Division of Medical Affairs, Zhongnan Hospital of Wuhan University, Wuhan, 430071 China; 12 Division of Nursing Affairs, Zhongnan Hospital of Wuhan University, Wuhan, 430071 China; 13 Office of Nosocomial Infection Control, Zhongnan Hospital of Wuhan University, Wuhan, 430071 China; 14 Emergency Center, Zhongnan Hospital of Wuhan University, Wuhan, 430071 China; 15 Department of Radiology, Zhongnan Hospital of Wuhan University, Wuhan, 430071 China; 16 Department of Health Research Methods, Evidence, and Impact, McMaster University, Hamilton, ON L8S 4L8 Canada; 17 Department of Traditional Chinese Medicine, Zhongnan Hospital of Wuhan University, Wuhan, 430071 China; 18 Global Health Institute, Wuhan University, Wuhan, 430072 China

**Keywords:** 2019 novel coronavirus, 2019-nCoV, Respiratory disease, Pneumonia, Infectious diseases, Rapid advice guideline, Clinical practice guideline, Evidence-based medicine

## Abstract

In December 2019, a new type viral pneumonia cases occurred in Wuhan, Hubei Province; and then named “2019 novel coronavirus (2019-nCoV)” by the World Health Organization (WHO) on 12 January 2020. For it is a never been experienced respiratory disease before and with infection ability widely and quickly, it attracted the world’s attention but without treatment and control manual. For the request from frontline clinicians and public health professionals of 2019-nCoV infected pneumonia management, an evidence-based guideline urgently needs to be developed. Therefore, we drafted this guideline according to the rapid advice guidelines methodology and general rules of WHO guideline development; we also added the first-hand management data of Zhongnan Hospital of Wuhan University. This guideline includes the guideline methodology, epidemiological characteristics, disease screening and population prevention, diagnosis, treatment and control (including traditional Chinese Medicine), nosocomial infection prevention and control, and disease nursing of the 2019-nCoV. Moreover, we also provide a whole process of a successful treatment case of the severe 2019-nCoV infected pneumonia and experience and lessons of hospital rescue for 2019-nCoV infections. This rapid advice guideline is suitable for the first frontline doctors and nurses, managers of hospitals and healthcare sections, community residents, public health persons, relevant researchers, and all person who are interested in the 2019-nCoV.

## 1 Background

In December 2019, the 2019 novel coronavirus (2019-nCoV) was discovered and identified in the viral pneumonia cases that occurred in Wuhan, Hubei Province, China; And then was named by the World Health Organization (WHO) on 12 January 2020. In the following month, the 2019-nCoV quickly spreading inside and outside of Hubei Province and even other countries. What’s more, the sharp increase of the case number caused widespread panic among the people.

Medical professionals require an up-to-date guideline to follow when an urgent healthcare problem emerging. In response to the requests for reliable advice from frontline clinicians and public healthcare professionals managing 2019-nCoV pandemics, we developed this rapid advance guideline, involving disease epidemiology, etiology, diagnosis, treatment, nursing, and hospital infection control for clinicians, and also for public health workers and community residents.

## 2 Guideline methodology

This guideline was prepared in accordance with the methodology and general rules of WHO Guideline Development and the WHO Rapid Advice Guidelines [1, 2].

### 2.1 Composition of the guideline development group

This guideline development group is multidisciplinary and composed of individuals from health professionals and methodologists. Health professionals included frontline clinical doctors, nurses who work in departments of respiratory medicine, fever clinic, critical medicine, emergency, infectious disease, and experts of respiratory infectious disease and hospital management board. The methodologists included methodologists of guideline development, systematic review, and literature searching professionals.

### 2.2 The end-user of the guideline

This guideline is suitable for frontline doctors and nurses, managers of hospitals and healthcare sections, healthy community residents, personnel in public healthcare, relevant researchers, and all persons who are interested in the 2019-nCoV management.

### 2.3 The target population of the guideline

This guideline is aimed to serve the healthcare professionals to tackle the suspected 2019-nCoV infected cases, confirmed 2019-nCoV infected cases, clustered 2019-nCoV infected cases, and those with close contacts or suspicious exposure to 2019-nCoV infected cases.

### 2.4 A survey of conflict of interests

Oral inquiry for financial interests of relevant personal was conducted at the first meeting while to start this guideline. Relevant financial as well as nonfinancial interests were surveyed and disclosed and subsequently assessed in consensus conference in order to minimize potential bias in guideline development. Finally, there is no conflict of interests for all the personnel participating to prepare this guideline.

### 2.5 Guideline’s structural setup and refining the topics and coverage of this guideline

This guideline is a rapid guideline to responding to the emerging infectious disease of 2019-nCoV. Due to the urgent need and tight work schedule, we conducted no wide-range survey but a discussion meeting with front-line clinicians who managed patients with 2019-nCoV infections to finalize guideline topics and key questions.

### 2.6 Literature searching and preparation of evidence profiles

#### 2.6.1 General notes

Considering the lack of direct evidence for this newly identified 2019-nCoV infection, we searched and referred to the guidelines that related to the SARS (Severe Acute Respiratory Syndrome), MERS (Middle East Respiratory Syndrome), and influenza. We also referred to the guidelines which were newly-issued by the National Health Commission of People’s Republic of China and WHO for 2019-nCoV. In addition, we have an independent literature searching team to search available indirect evidence from systematic reviews and/or RCTs (randomized controlled trials), which were for the treatment and/ or chemoprophylaxis of SARS, MERS, or other influenza virus infections.

If the existing evidence addressed topics or questions covered by the guideline, then its quality should be assessed. If there is a lack of higher-level quality evidence, our panel considered observational studies and case series. Because of the limited time, we did not perform new systematic review. We identified relevant literature up to 20 January 2020.

#### 2.6.2 Search resources

We searched the bibliographic databases: PubMed, Embase, and Cochrane library.

We also searched following websites: the WHO (https://www.who.int/), CDC (Centers for Disease Control and Prevention, https://www.cdc.gov/), NICE (National Institute for Health and Clinical Excellence, https://www.nice.org.uk/), National Health Commission of the People’s Republic of China (http://www.nhc.gov.cn/), and National Administration of Traditional Chinese Medicine (http://www.satcm.gov.cn/).

#### 2.6.3 Frontline data collection and summary

As the 2019-nCoV is a newly identified pathogen responsible for the outbreak of the pandemic disease, there is no sufficient evidence to reveal the whole nature of this virus. In these situations, obtaining evidence from the experts who fighting the disease on the frontline can be efficient and the main source [3].

Until to 24:00 on 29 January 2020, 11,500 persons were screened, and 276 were identified as suspected infectious victims, and 170 were diagnosed (including 33 in critical condition) for 2019-nCoV infection in Zhongnan Hospital of Wuhan University. During this process, frontline clinicians and nurses have accumulated valuable experience in the diagnosis, treatment and nursing for 2019-nCoV infected patients. Hence, these experiences were assessed and then used as “Expert Evidence” for our guideline development. We took interviews and group surveys to collect information on treatment evidence during the guideline panel’s meeting, so that it could be integrated into the guideline panel in the summary of findings (see Additional files 1 and 2). Experts’ evidence can be solicited by the description of case reports, summaries, and reports of topics or questions of all cases they management.

### 2.7 Grading the evidences and recommendations

We accorded to the Grading of Recommendations Assessment, Development and Evaluation (GRADE) basic approaches and rules [4, 5], and particularly considered experts’ evidence to assess the quality of a body of evidence to make recommendations.

The quality of evidence reflects whether the extent to which our confidence estimating the effect is adequate to support a particular recommendation. The level of evidence was categorized as “high quality”, “moderate quality”, “low quality”, or “very low quality”; Recommendations were classified as “strong” or “weak.”

The strong recommendation does not always mean there is sufficient intervention effectiveness. Besides the effectiveness of intervention, the forming of recommendations is based on the severity of the disease, patient willingness, safety, and economics [4]. See Tables 1 and 2 [4, 6].

**Table 1:** Classification and description of recommendation

Classification of recommendation	Description
Strong recommendation	It is definite that the desirable effects of an intervention outweigh its undesirable effects or the undesirable effects of an intervention outweigh its desirable effects
Weak recommendation	The desirable effects probably outweigh the undesirable effects or undesirable effects probably outweigh the desirable effects

**Table 2:** Rules for grading the recommendations

Strength of recommendation and quality of evidence	Benefit *vs.* risk and burdens	Methodological quality of supporting evidence^a^	Implications
Strong recommendation, high-quality evidence	Benefits clearly outweigh risk and burdens, or vice versa	RCTs without important limitations or overwhelming evidence from observational studies	Strong recommendation, can apply to most patients in most circumstances without reservation
Strong recommendation, moderate quality evidence	Benefits clearly outweigh risk and burdens, or vice versa	RCTs with important limitations (inconsistent results, methodological flaws, indirect or imprecise) or exceptionally strong evidence from observational studies	Strong recommendation, can apply to most patients in most circumstances without reservation
Strong recommendation, low or very low quality evidence	Benefits clearly outweigh risk and burdens, or vice versa	Observational studies or case series	Strong recommendation but may change when higher quality evidence becomes available
Weak recommendation, high-quality evidence	Benefits closely balanced with risks and burden	RCTs without important limitations or overwhelming evidence from observational studies	Weak recommendation, best action may differ depending on circumstances or patients’ or societal values
Weak recommendation, moderate quality evidence	Benefits closely balanced with risks and burden	RCTs with important limitations (inconsistent results, methodological flaws, indirect or imprecise) or exceptionally strong evidence from observational studies	Weak recommendation, best action may differ depending on circumstances or patients’ or societal values
Weak recommendation, low or very low quality evidence	Uncertainty in the estimates of benefits, risks and burden; benefits, risk and burden may be in a closely balanced	Observational studies or case series	Very weak recommendations; other alternatives may be equally reasonable

*RCTs* randomized controlled trials

^a^The evidence agreed on by more than 70% frontline clinicians in consensus meeting is viewed as high-quality evidence

### 2.8 Forming the recommendations

Before meetings, experts’ evidence was collected systematically and available to panel members. Once the evidence has been identified and assessed, recommendations were formulated based on the evidence by a face-to-face meeting of panel members and supplemented by experts participating in the panel meeting.

Experts’ evidence was highly valued in this guideline development. During the consensus process, if the evidence was agreed on by more than 70% frontline clinicians in the consensus meeting, it is considered as high-quality evidence.

In specific recomendations, we used “should” or “strongly recommend” for strong recommendations; whereas, “suggest” or “consider” was used for weak ones.

### 2.9 Drafting and publishing the guideline

This guideline was published in both Chinese and English versions at the same time. Due to space limitations, the current standard revision does not include evidence descriptions. The full revision will be published in *New Medicine* (Chinese name: Yixue Xinzhi; http://www.jnewmed.com/), Volume 30 and Issue 1 2020 [7].

## 3 Epidemiological characteristics

### 3.1 Scope of the 2019-nCoV infection outbreak

Since December 2019, multiple cases occurring unexplainable pneumonia were successively reported in some hospitals in Wuhan city with a history of exposure to a large Hua’nan seafood market in Wuhan city, Hubei province, China. It has been confirmed to be an acute respiratory infection caused by a novel coronavirus. So far, the number of cases without a history of the Hua’nan seafood market exposure is increasing. In addition, clustered cases and confirmed cases without a history of travel to Wuhan emerged. Also, confirmed cases without clear exposure to the Wuhan seafood market have been found in many foreign countries or regions [8].

At 24:00 on 26 January 2020, the National Health Commission of the People’s Republic of China has recorded a total of 2744 confirmed cases of pneumonia with 2019-nCoV infection from 30 provinces (districts and cities), including 461 severe cases and 80 deaths. A total of 51 cases have been cured and discharged. At present, 5794 suspected cases were recorded, 32,799 with close contacts to the confirmed patients have been tracked, 583 people were released from medical observation that day, and 30,453 people were still undergoing medical observation. A total of confirmed cases were reported from Hong Kong, Macao and Taiwan of China: 8 cases in Hong Kong, 5 cases in Macao, and 4 cases in Taiwan. In addition, confirmed cases had been reported from abroad: 7 in Thailand, 4 in Australia, 4 in Singapore, 3 in France, 3 in Japan, 3 in Korea, 3 in Malaysia, 3 in the United States, 2 in Vietnam, and one in Nepal [9].

### 3.2 Host and reservoir

Wild animal, bats [10] is the most possible host of the 2019-nCoV. It requires further confirmation whether pneumonia infected by the 2019-nCoV is transmitted directly from bats or through an intermediate host. It is believed that clarifying the source of the virus will help determine zoonotic transmission patterns [11].

### 3.3 Route of transmission

Up to present, the main infection source was the patients who with pneumonia infected by the 2019-nCoV. Respiratory droplet transmission is the main route of transmission, and it can also be transmitted through contact [12]. Although many details, such as the source of the virus and its ability to spread between people remain unknown, an increasing number of cases show the signs of human-to-human transmission [8, 13].

### 3.4 Etiology and pathogenesis

The 2019-nCoV isolated from the lower respiratory tract of patients with unexplainable pneumonia in Wuhan, and it is a novel coronavirus belonging to the β genus. The 2019-nCoV has an envelope; its particles are round or oval, often polymorphic, with a diameter from 60 nm to 140 nm. Its genetic characteristics are significantly different from SARSr-CoV (SARS related coronaviruses) and MERSr-CoV (MERS related coronaviruses). Current research shows it has more than 85% homology with SARSr-CoV (bat-SL-CoVZC45). 2019-nCoV can be found in human respiratory epithelial cells 96 h after in vitro isolation and culture, while it takes about 6 days in VeroE6 or Huh-7 cell lines [12].

The source of the virus, the time span of the patients discharging infective virus, and the pathogenesis are still not clear [14].

### 3.5 Molecular epidemiology

No evidence of viral mutation has been found so far [14]. It is necessary to obtain much more clinically isolated viruses with time and geographical variety to assess the extent of the virus mutations, and also whether these mutations indicate adaptability to human hosts [11].

### 3.6 Incubation and contagious period

Based on currently epidemiological survey, the latency period is generally from 3 to 7 days, with a maximum of 14 days [10]. Unlike SARSr-CoV, 2019-nCoV is contagious during the latency period [15].

### 3.7 Prognostic factors

The population is generally susceptible to the virus. The elderly and those with underlying diseases show more serious conditions after infection, and children and infants also get infected by the 2019-nCoV. From current knowledge of the cases, most patients have a good prognosis, the symptoms of children are relatively mild, and a few patients are in critical condition. Death cases are more frequently seen in the elderly and those with chronic underlying diseases [12].

The newest study including the first 41 confirmed cases admitted to Wuhan between 16 December 2019 and 2 January 2020 showed the median age of patients was 49 years; and the main underlying diseases were diabetes, hypertension, and cardiovascular diseases. Of them, 12 cases experienced acute respiratory distress syndrome (ARDS), 13 cases were admitted to the intensive care unit (ICU), and 6 cases died [16].

## 4 Screening for diseased cases and preventive measures for population

### 4.1 Case definitions

#### 4.1.1 Suspected case

Patients with any 2 of the following clinical features and any epidemiological risk: (1) clinical features: fever, imaging features of pneumonia, normal or reduced white blood cell count, or reduced lymphocyte count in the early stages of the disease onset. (2) epidemiologic risk: a history of travel to or residence in Wuhan city, China or other cities with continuous transmission of local cases in the last 14 days before symptom onset; contact with patients with fever or respiratory symptoms from Wuhan city, China or other cities with continuous transmission of local cases in the last 14 days before symptom onset; or epidemiologically connected to 2019-nCoV infections or clustered onsets [12].

#### 4.1.2 Confirmed case

Those with one of the following pathogenic evidence is the confirmed case: (1) positive for the 2019-nCoV by the real-time PCR test for nucleic acid in respiratory or blood samples [17]. 2) viral gene sequencing shows highly homogeneity to the known 2019-nCoV in respiratory or blood samples [12].

#### 4.1.3 Clustered cases

Suspected clustering cases are defined when one confirmed case and at the same time, one or more cases of fever or respiratory infection are found in a small area (such as a family, a construction site, a unit, etc.) within 14 days.

Under the above circumstances, 2 or more confirmed cases are found, and there is the possibility of human-to-human transmission due to close contact or infection due to co-exposure, then it is determined as a clustered case [8, 18].

#### 4.1.4 Close contacts

Those who have one of the following contacts after the onset of confirmed cases in the absence of effective protection [18]: (1) those who live, study, work, or have close contact with the confirmed cases, or other close contacts such as working closely with or sharing the same classroom or living in the same house with the confirmed case. (2) medical and nursing staffs and their family members living with them, who treated, nursed or visited the confirmed case, or other personnel who have similar close contact with the case, such as providing direct treatment or care of the case, visiting the case or staying in a closed environment where the cases are located; other patients or caregivers in the same room with the case. (3) people who have close contact with the patients in a same transport vehicle, including those who had taken care of the patients on the vehicle; the person who had companied the patients (family members, colleagues, friends, etc.); other passengers and traffic staff considered having likely close contact with the patients by investigation and evaluation. (4) other circumstances considered to be closely contacted with the person with close contact with the patients by the professional investigation and evaluation.

#### 4.1.5 Suspicious exposure

Persons with suspicious exposure are those who are exposed without effective protection to processing, sales, handling, distributing, or administrative management of wild animals, materials, and the environments that are positive for the 2019-nCoV test [18].

### 4.2 Prevention

#### 4.2.1 Persons with close contacts and suspicious exposure

Persons with close contacts and suspicious exposure should be advised to have a 14-day health observation period, which starts from the last day of contact with the 2019-nCoV infected patients or suspicious environmental exposure. Once they display any symptoms, especially fever, respiratory symptoms such as coughing, shortness of breath, or diarrhea, they should reach out for medical attention immediately [19]. Contact surveillance should be carried out for those who had exposed to accidental contact, low-level exposure to suspected or confirmed patients, i.e. checking any potential symptoms when carrying out daily activities [20]. See Table 3 for details [21].

**Table 3:** Recommendations for those with close contacts and suspicious exposures

No.	Recommendation items	Recommendation strength
1	Strictly take the observation period of 14 days, and go to the hospital for diagnosis and treatment if symptoms appear (fever, cough, etc.).	Strong
2	If available, inform the designated hospital in advance to send cars to pick up the patients with symptoms to the hospital.	Weak
3	Patients should wear N95 masks (priority strategy).	Strong
4	Using disposable surgical mask (alternative strategy).	Weak
5	Avoid taking public transportation to the hospital, choose an ambulance or private vehicle, and open vehicle windows for ventilation on the way to the hospital (priority strategy).	Strong
6	When walking on the road or waiting in the hospital, try to stay away from other people (at least 1 m away) and wear a mask.	Strong
7	The family members accompanying those for inspection should immediately follow the monitoring recommendations to close contacts, keep the respiratory hygiene and clean their hands properly.	Strong
8	The community or street hospital should be informed before the suspected contacts to the hospital. The vehicle used should be cleaned and disinfected with 500 mg/L chlorine-containing disinfectant, and the window should be opened for ventilation.	Strong

#### 4.2.2 Patients with suspected 2019-nCoV infection

Patients with a suspected infection should be isolated, monitored, and diagnosed in hospital as soon as possible. Doctors should make recommendations based on the patient’s situation. Patients with mild symptoms and suspected infection may consider in-home isolation and home care (*weak recommendation*). Suspected infected with severe symptoms and those who need to stay in hospital for observation by doctor’s judgment should follow the isolation guidelines for suspected patients (see Tables 4 and 5 for details).

**Table 4:** Criteria to define patients with suspected mild symptoms

No.	Definition of suspected patients with mild symptoms
1	In-home isolation and care after assessment by doctor (golden standard)
2	With a fever < 38 ℃
3	The fever can go down by itself
4	No dyspnea, no asthma
5	With or without cough
6	No underlying chronic diseases, *e.g.*: heart, lung and kidney diseases

**Table 5:** Home care and isolation guidelines for suspected patients with mild symptoms

No.	Recommendation items	Recommendation strength
Suspected patients with mild symptoms		
1	Well-ventilated single rooms (preferred strategy).	Strong
2	Maintain a bed distance of at least 1 m from the patient (alternative strategy).	Weak
3	Clean and disinfect household articles using 500 mg/L chlorine-containing disinfectant frequently every day (wide range).	Strong
4	Limit visits by relatives and friends.	Strong
5	The caregiver should be a healthy family member without underlying diseases.	Weak
6	Restrict the patient’s activity	Strong
7	Open windows for ventilation in shared areas such as toilets and kitchens.	Strong
8	Avoid sharing toothbrush, towel, tableware, bed sheet and other items with patients. The patient’s daily necessities are for single use only and should be placed separately from that of their family members.	Strong
9	When coughing or sneezing, it is necessary to wear a medical mask, or cover with a paper towel and bent elbow, and clean hands immediately after coughing and sneezing.	Strong
10	N95 masks should be worn in the same room with patients (preferred strategy).	Strong
11	Disposable surgical mask (alternative strategy). Use the mask in strict accordance with the instruction manual.	Weak
12	After washing hands with running water, dry them with a paper towel (preferred strategy).	Strong
13	Dry with a towel, and wash and disinfect the towel daily (alternative strategy).	Weak
Home caregivers		
1	Clean and disinfect hands after contact with the patient, before leaving patient’s room or the house, before and after eating, after using the toilet and after entering house from outside (for visible contaminant on hands, wash hands with running water then use hand disinfection).	Strong
2	Avoid direct contact with patient’s secretions or discharges, especially oral or respiratory discharges; avoid direct contact with patient’s feces.	Strong
3	Wear disposable gloves (double layers) when providing oral and respiratory care to patients, handling patient’s feces and urine, and cleaning the patient’s room, etc. Wash hands before wearing gloves and after removing the gloves.	Strong
4	Wash the patient’s clothes, bed sheets, bath towels, towels, etc. with ordinary washing soap and water, or use a washing machine at 60–90 ℃ with ordinary household washing liquid (Strong recommendation), or routinely wash them with washing machine after soaking in low concentration disinfectant (Weak recommendation).	Strong/Weak
5	Put the contaminated bedding into the laundry bag. Do not shake contaminated clothing and avoid direct contact.	Strong
6	The waste generated by the patient should be put into the closed garbage bags and replaced frequently.	Strong

It should also be noted that: (1) whether the suspected patients should be given in-home isolation and care or not requires careful clinical evaluation and safety assessment by professionals. (2) if the suspected patients do not get improvement in the symptoms or even worsened in condition during home care, they need to go to the doctor for treatment. (3) during the period of home care, the patients’ medication and symptoms should be closely recorded and their caregivers should also monitor their body temperature daily.

Throughout the period of home care, healthcare personnel should perform regular (*e.g.*, daily) follow-up through face-to-face visits or phone interviews (ideally, if feasible) to follow the progress of symptoms and, if necessary, specific diagnostic tests should be conducted [14, 19, 21].

#### 4.2.3 Prevention for travellers (*Strong recommendation*)

International visitors should take routine precautions when entering and leaving the affected areas, including avoiding close contacts with people with acute respiratory infection, washing hands frequently, especially after contacting with the sick or their surrounding environment; following appropriate coughing etiquette; and avoiding close contact with live or dead farming animals or bats or other wild animals [22, 23]. Passengers should avoid unnecessary travel as possible.

If they had travelled to Hubei Province (especially Wuhan city) in the past 14 days and is in fever, cough or difficulty in breathing, they should: (1) see a doctor immediately; (2) call the doctor about his/her recent trips and symptoms before going to the doctor’s office or emergency room; (3) avoid contact with others; (4) not to travel around; (5) cover mouth and nose with a tissue or sleeve (not hands) when coughing or sneezing; and (6) wash hands with soap and water for at least 20 s. If soap and water are not available, use alcohol-based hand sanitizers [24].

## 5 Diagnosis of the 2019-nCoV cases

### 5.1 Clinical manifestation

The 2019-nCoV infected cases have symptoms like fever, fatigue, dry cough, dyspnea etc., with or without nasal congestion, runny nose or other upper respiratory symptoms [13, 16]. Despite the atypical symptoms were reported [25], Nan-Shan Zhong, the academician of Chinese Academy of Engineering in an exclusive interview with Xinhua News Agency on 28 January 2020, pointed out that fever is still the typical symptom of 2019-nCoV infection.

### 5.2 Physical examination

Patients with mild symptoms may not present positive signs. Patients in severe condition may have shortness of breath, moist rales in lungs, weakened breath sounds, dullness in percussion, and increased or decreased tactile speech tremor, etc.

### 5.3 Imaging examination

#### 5.3.1 CT imaging (*strong recommendation*)

The imaging findings vary with the patient’s age, immunity status, disease stage at the time of scanning, underlying diseases, and drug interventions.

The imaging features of lesions show: (1) dominant distribution (mainly subpleural, along the bronchial vascular bundles); (2) quantity (often more than three or more lesions, occasional single or double lesions); (3) shape (patchy, large block, nodular, lumpy, honeycomb-like or grid-like, cord-like, etc.); (4) density (mostly uneven, a paving stones-like change mixed with ground glass density and interlobular septal thickening, consolidation and thickened bronchial wall, etc.); and (5) concomitant signs vary (air-bronchogram, rare pleural effusion and mediastinal lymph nodes enlargement, etc.).

#### 5.3.2 Clinical data from Zhongnan Hospital of Wuhan University

##### Typical CT/X-ray imaging manifestation, including

**Fig. 1:**
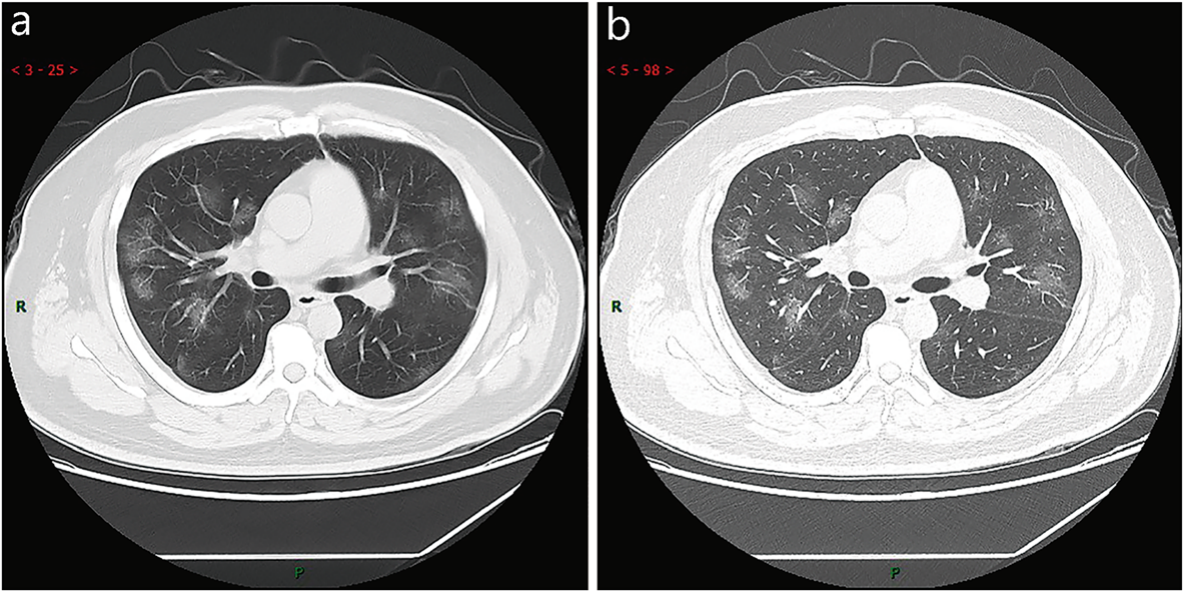
Typical CT imaging manifestation (case 1). A 38 years old male with fever without obvious inducement (39.3 ℃), dry cough and shortness of breath for 3 days. Laboratory test: normal white blood cells (6.35 × 10^9^/L), decreased lymphocytes percentage (4.1%), decreased lymphocytes count (0.31 × 10^9^/L), decreased eosinophil count (0 × 10^9^/L)), increased C-reaction protein (170.91 mg/L), increased procalcitonin (0.45 ng/ml). Imaging examination: multiple patches, grid-like lobule and thickening of interlobular septa, typical “paving stone-like” signs. **a** SL(Slice): 6 mm; **b** high-resolution computed tomography(HRCT). HRCT. high-resolution computed tomography

**Fig. 2:**
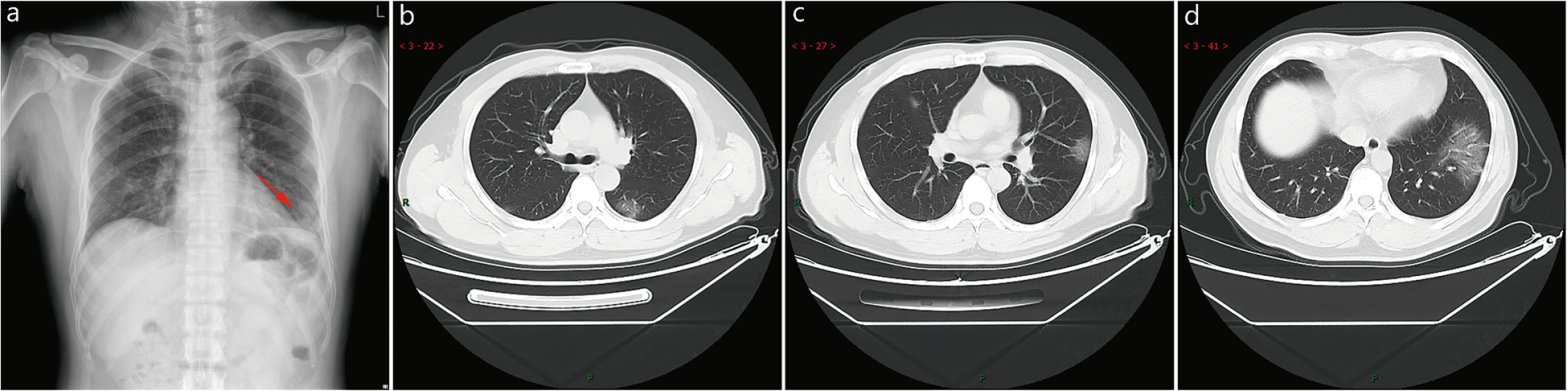
Typical CT / X-ray imaging manifestation (case 2). A 51 years old male with general muscle ache and fatigue for 1 week, fever for 1 day (39.1 ℃), anemia. Laboratory test: normal white blood cells (9.24 × 10^9^/L), lymphocytes percentage (5.1%), decreased lymphocytes (0.47 × 10^9^/ L), decreased eosinophil count (0 × 10^9^/L), increased C-reaction protein (170.91 mg/L), increased procalcitonin (0.45 ng/ml), increased erythrocyte sedimentation rate (48 mm/h). Imaging examination: **a** shows patchy shadows in the outer region of the left lower lobe, **b** shows large ground-glass opacity in the left lower lobe, and **c** shows subpleural patchy ground-glass opacity in posterior part of right upper lobe and lower tongue of left upper lobe, **d** shows large ground-glass opacity in the basal segment of the left lower lobe

**Fig. 3:**
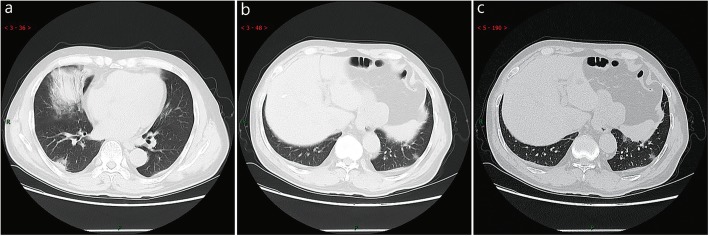
Typical CT / X-ray imaging manifestation (case 3). A 65 years old male with fever for 4 days (38.7 ℃). Laboratory test: normal white blood cells (3.72 × 10^9^/L), decreased lymphocytes (0.9 × 10^9^/ L), decreased eosinophil count (0 × 10^9^/L), increased C-reaction protein (53.0 mg/L), increased procalcitonin (0.10 ng/ml), reduced liver function, hypoproteinemia, and mild anemia. Imaging examination: **a** and **b** showed large consolidation in the right middle lobe, patchy consolidation in the posterior and basal segment of the right lower lobe, with air-bronchogram inside, **c** showed patchy consolidation in the outer and basal segment of the left lower lobe, and a small amount of effusion in the right chest

1. Multiple, patchy, sub-segmental or segmental ground-glass density shadows in both lungs. They were classified as “paving stone-like” changes by fine-grid or small honeycomb-like thickening of interlobular septa. The thinner the CT scan layers, the clearer the ground-glass opacity and thickening of interlobular septa were displayed. A slightly high-density and ground-glass change with fuzzy edge in the fine-grid or small honeycomb-like thickening of interlobular septa were presented by the high-resolution computed tomography (HRCT), (Fig. 1: 45 cases, 54.2% in a total of 83 cases). The resolution of X-ray was worse lower than that of CT in the resolution, which was basically manifested as ground-glass opacities with fuzzy edge (Fig. 2: 9 cases, 10.8% in a total of 83 cases).

(2) Multiple, patchy or large patches of consolidation in both lungs, with a little grid-like or honeycomb-shaped interlobular septal thickening, especially in the middle and lower lobes (Fig. 3: 26 cases, 31.3% in a total of 83 cases). It was more common in the elderly or severe condition patients.

##### Atypical CT/X-ray imaging manifestation, including

**Fig. 4:**
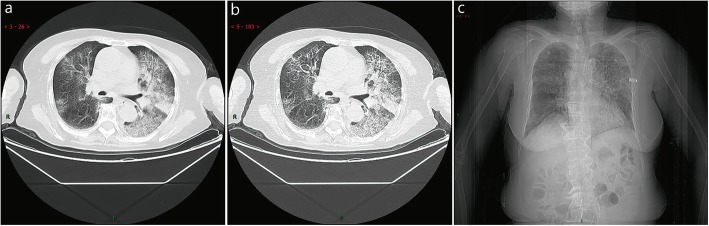
Atypical CT / X-ray imaging manifestation (case 1). An 83 years old female with fever for 4 days (maximum temperature of 38.8 ℃), cough, chills, sore throat, dry cough for 1 week, chest tightness and shortness of breath aggravating for 1 week. Laboratory test: normal white blood cells (4.6 × 10^9^/L), normal neutrophil percentage (65.8%), decreased lymphocytes percentage (19.9%). Imaging examination: **a** and **b** showed diffuse interlobular septum thickening in both lungs to form a grid opacity, thickening of bronchial wall, and consolidation in the left sublobal lung. **c** showed diffused grid-like opacities in both lungs, especially in the left lung

**Fig. 5:**
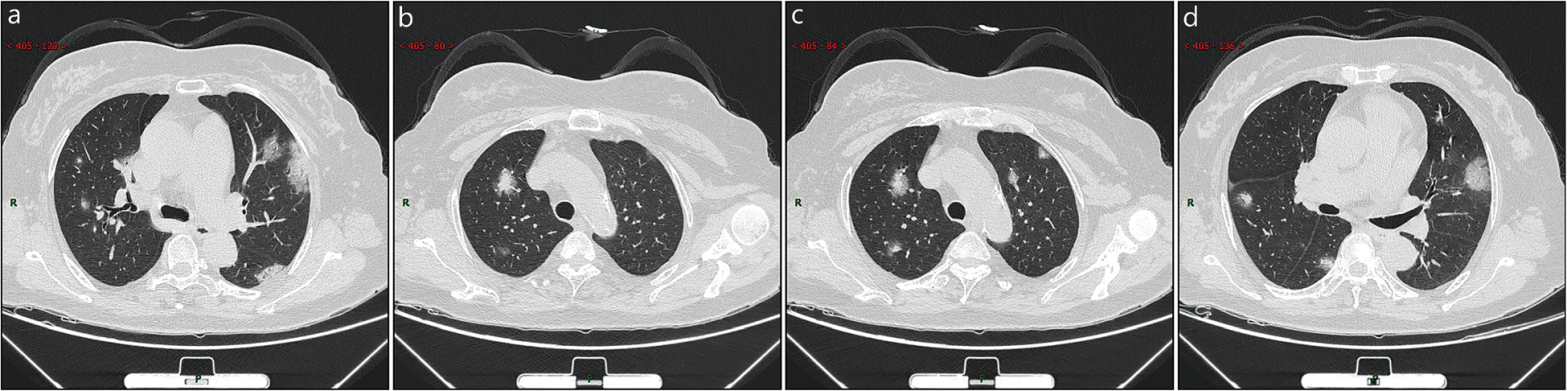
Atypical CT / X-ray imaging manifestation (case 2). A 56 years old female with fever for 3 days. Laboratory test: decreased total protein (54.0 g/L),decreased albumin (35.5 g/L),decreased globulin (18.5 g/L), normal white blood cells (4.87 × 10^9^/L), decreased lymphocytes percentage (10.1%), decreased lymphocytes (0.49 × 10^9^/ L), decreased eosinophil count (0 × 10^9^/L)), decreased eosinophil count percentage (0%). Imaging examination: **a** showed two consolidation nodulesat the center of the lateral segment of middle lobe of the right lung which was surrounded by ground-glass opacities; **b** showed patchy ground-glass opacity in the anterior segment of the right upper lung with patchy consolidation lesions in it; **c** showed patchy ground-glass opacities in both lungs with patchy consolidation lesions in it. **d** showed patchy consolidation in the ground-glass opacities in the middle lobe and dorsal segment of lower lobe of right lung

1. Single, or multiple, or extensive subpleural grid-like or honeycomb-like thickening of interlobular septum, thickening of the bronchial wall, and tortuous and thick strand-like opacity. Several patchy consolidations, occasionally with a small amount pleural effusion or enlargement of mediastinal lymph nodes, can be seen (Fig. 4: 6 cases, 7.2% in a total of 83 cases). This is mostly seen in the elderly.

(2) Single or multiple solid nodules or consolidated nodules in the center of lobule, surrounded by ground-glass opacities (Fig. 5: 5 cases, 6.2% in a total of 83 cases).

##### Stage based on CT image

**Fig. 6:**
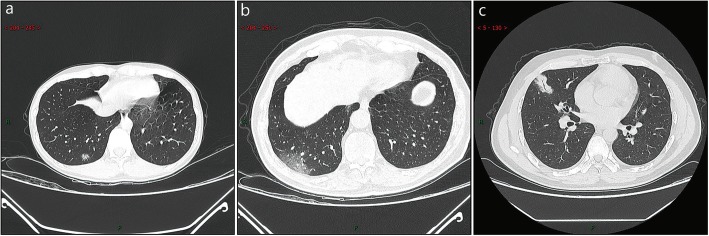
CT imaging of ultra-early stage. **a** A 33 years old female with patchy ground-glass opacities after occupational exposure. **b** A 67 years old male with a history of contact with infected patients, showing large ground-glass opacity. **c** A 35 years old female exhibiting large consolidated opacity with air-bronchogram inside after occupational exposure

**Fig. 7:**
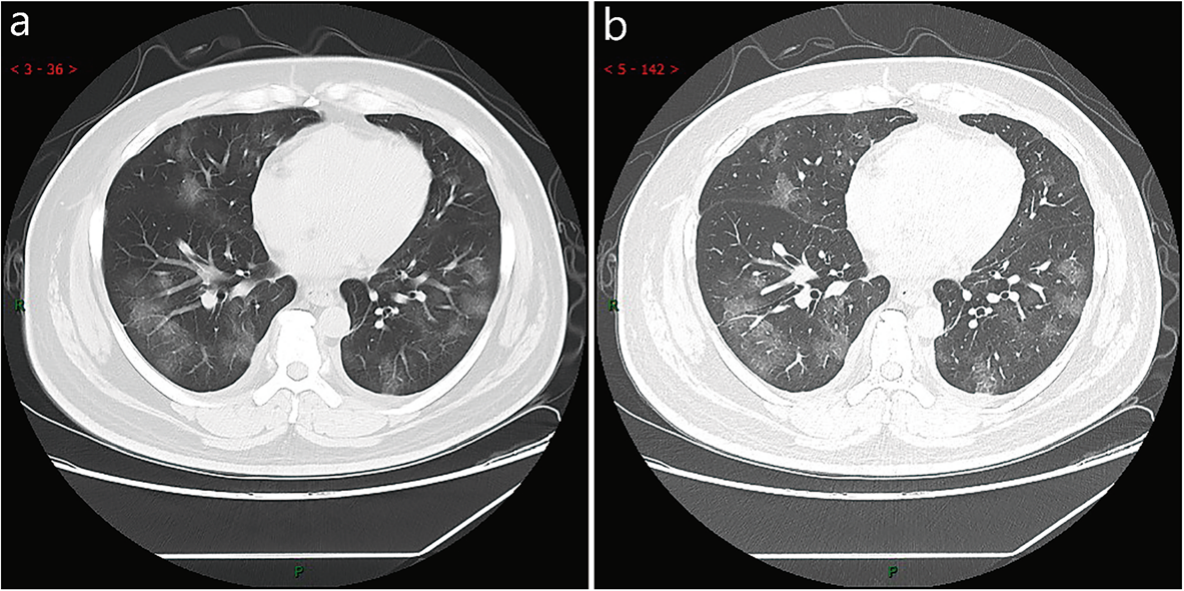
CT imaging of early stage. Male, 38 years old, fever without obvious inducement (39.3 ℃), dry cough and shortness of breath for 3 days. Laboratory test: decreased white blood cells (3.01 × 10^9^/L), decreased lymphocytes (0.81 × 10^9^/ L), increased C-reaction protein (60.8 mg/L), increased procalcitonin (0.16 ng/ml). Imaging examination: **a** (thin layer CT) and **b** (high-resolution CT) showed multiple patchy and light consolidation in both lungs and grid-like thickness of interlobular septa

**Fig. 8:**
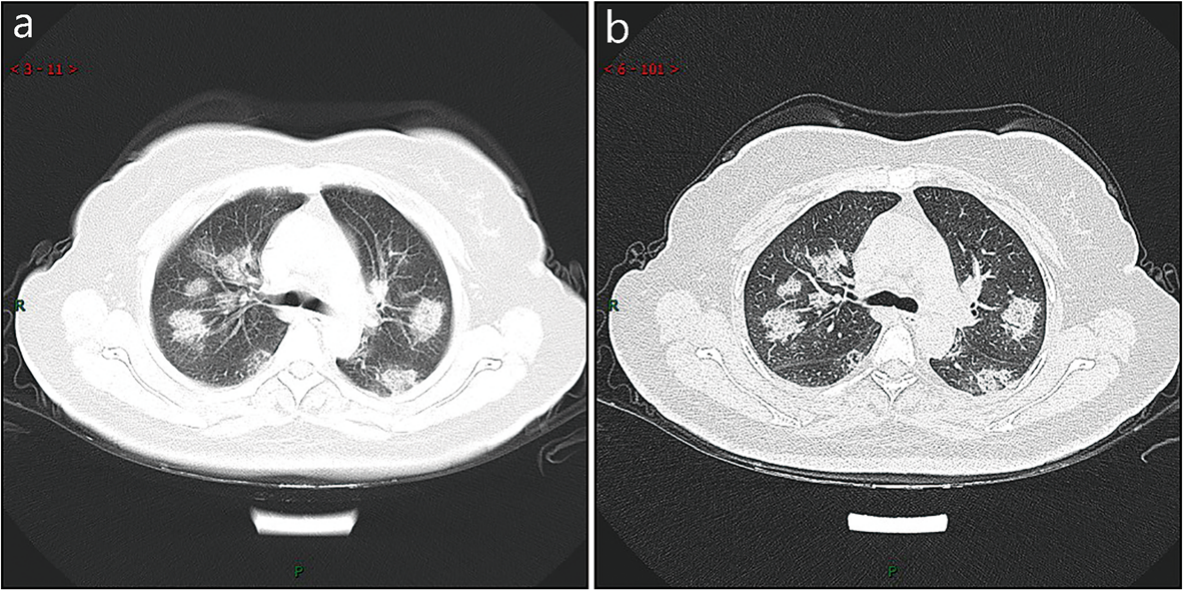
CT imaging of rapid progression stage. A 50 years old female with anorexia, fatigue, muscle soreness, nasal congestion and runny nose for 1 week, sore and itching throat for 2 days. Laboratory test: increased erythrocyte sedimentation rate (25 mm/h), normal white blood cells (4.08 × 10^9^/L), decreased lymphocytes (0.96 × 10^9^/ L), increased C-reaction protein (60.8 mg/L). Imaging examination: **a** (thin layer CT) and **b** (high-resolution CT) showed multiplepatchy and light consolidation in both lungs and grid-like thickness of interlobular septa

**Fig. 9:**
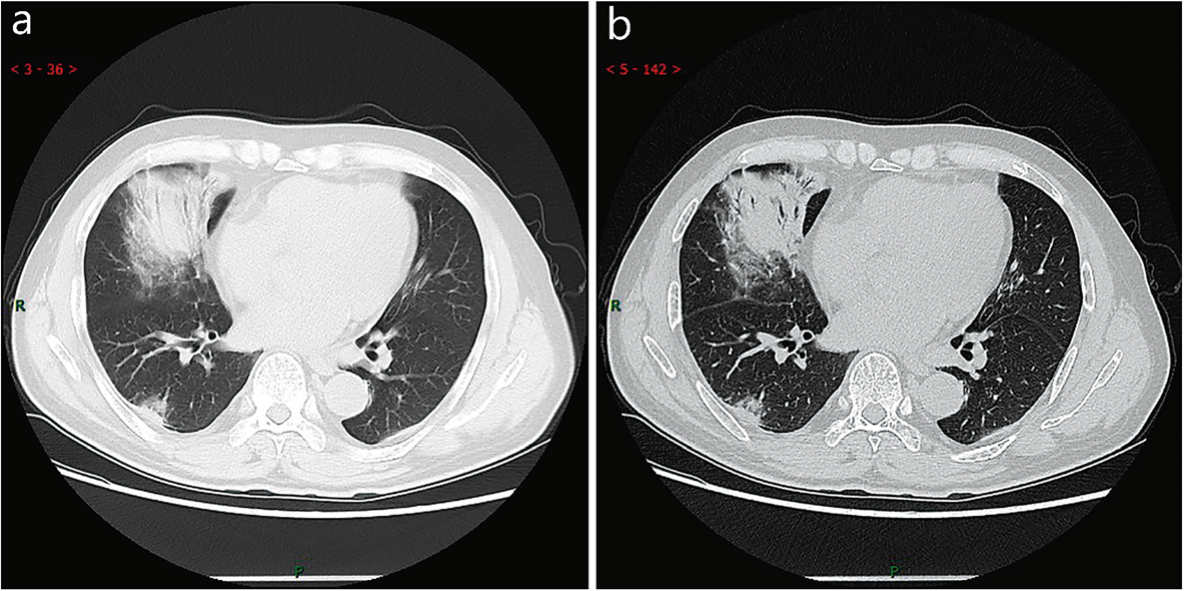
CT imaging of consolidation stage. A 65 years old male with fever (maximum temperature of 39 ℃). Laboratory test: hypoproteinemia (decreased total protein (62.20 g/L), decreased albumin (35.70 g/L)), abnormal liver function (increased alanine aminotransferase (79 U/L), increased aspartate aminotransferase (72 U/L)), increased procalcitonin (0.10 ng/ml), increased C-reaction protein (53 mg/L), decreased white blood cells (3.72 × 10^9^/L), decreased lymphocytes (0.9 × 10^9^/ L), mildanemia (decreased red blood cells (4.10 × 10^12^/L), decreased hemoglobin (131.10 g/L), decreased hematocrit (39.0%). Imaging examination: **a** (thin layer CT) and **b** (high-resolution CT) showedmultiple patchyand large consolidation in right middle lobe, posterior and basal segment of right lower lobe and outer and basal segment of left lower lobe, with air-bronchogram inside

**Fig. 10:**
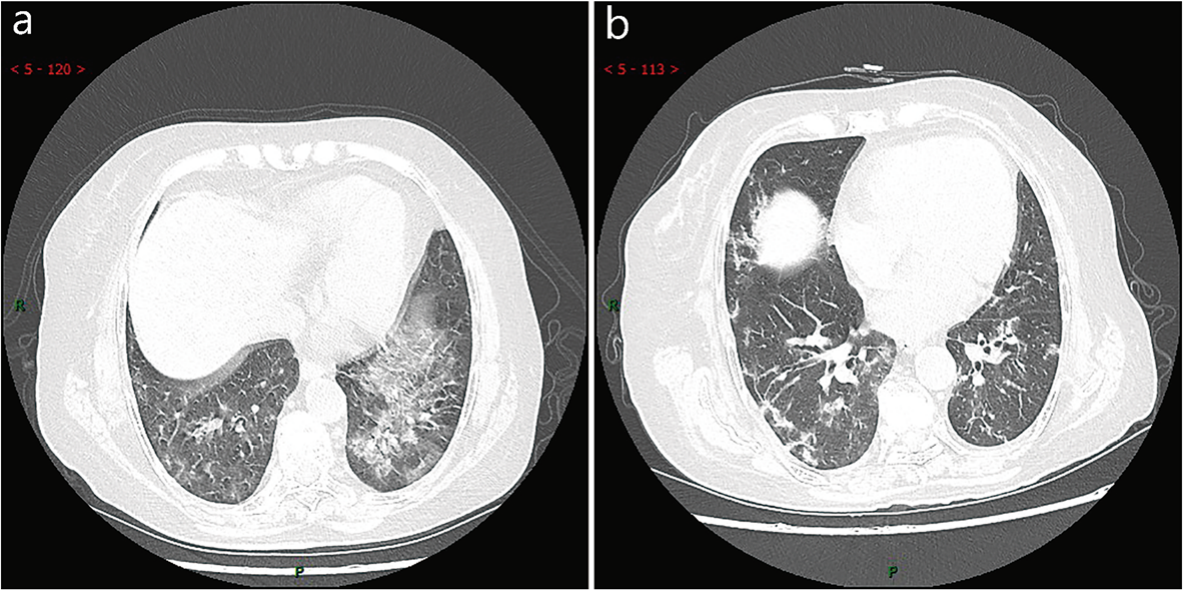
CT imaging of dissipation stage. A 79 years old female with intermittent fever. Laboratory test after 3 days of comprehensive treatment: decreased red blood cells (3.73 × 10^12^/L), hemoglobin (107 g/L), decreased hematocrit (31.8%), decreased lymphocytes percentage (13.9%), decreased lymphocytes (0.62 × 10^9^/ L), decreased eosinophil count percentage (0%), decreased eosinophil count (0 × 10^9^/L), increased alanine aminotransferase (46 U/L), deceased total protein (56.8 g/L), decreased albumin (33.5 g/L), normal C-reaction protein and procalcitonin. Imaging examination: **a** patchy ground-glass opacity and grid-like thickening of interlobular septa in the tongue-like segment of left upper lobe, and patchy consolidation in the posterior segment of right middle and lower lobe. **b** 9 days after admission to hospotial, CT scan showed absorption of lesions in the middle lobe, narrowing of lesions in the lower lobe of the right lung, and absorption of lesions in the tongue-like segment of left upper lobe which exhibited a cord-like change

The CT imaging demonstrates 5 stages according to the time of onset and the response of body to the virus, including:

1. Ultra-early stage. This stage usually refers to the stage of patients without clinical manifestation, negative laboratory test but positive throat swab for 2019-nCoV within 1–2 weeks after being exposed to a virus-contaminated environment (history of contact with a patient or patient-related family members, unit, or medical staff in a cluster environment). The main imaging manifestations are single, double or scattered focal ground-glass opacity, nodules located in central lobule surrounded by patchy ground-glass opacities, patchy consolidation and sign of intra-bronchial air-bronchogram, which was dominant in the middle and lower pleura (Fig. 6: 7 cases, 8.4% in a total of 83 cases).

(2) Early stage.This stage refers to the period of 1–3 days after clinical manifestations (fever, cough, dry cough, etc.). The pathological process during this stage is dilatation and congestion of alveolar septal capillary, exudation of fluid in alveolar cavity and interlobular interstitial edema. It showed that single or multiple scattered patchy or agglomerated ground-glass opacities, separated by honeycomb-like or grid-like thickened of interlobular septa (Fig. 7: 45 cases, 54.2% in a total of 83 cases).

(3) Rapid progression stage. This stage refers to the period about 3–7 days after clinical manifestations started, the pathological features in this stage are the accumulation of a large number of cell-rich exudates in the alveolar cavity, vascular expansion and exudation in the interstitium, both of which lead to further aggravation of alveolar and Interstitial edema. The fibrous exudation connects each alveolus through the inter-alveolar space to form a fusion state. The CT manifested a fused and large-scale light consolidation with air-bronchogram inside (Fig. 8: 17 cases, 20.5% in a total of 83 cases).

(4) Consolidation stage. This stage refers to the period around 7–14 days after clinical manifestations appeared. The main pathological features in this stage are the fibrous exudation of the alveolar cavity and the disappearance of capillary congestion in the alveolar wall. CT imaging showed the multiple patchy consolidations in slighter density and smaller range than that of the previous stage. (Fig. 9: 26 cases, 31.2% in a total of 83 cases).

(5) Dissipation stage. This stage refers to the period roughly between 2 and 3 weeks after the onset of clinical manifestations. The range of lesions was further reduced. CT imaging showed patchy consolidation or strip-like opacity. As time goes on, it showed grid-like thickening of interlobular septum, thickening and strip-like twist of bronchial wall and a few scattered patchy consolidations (Fig. 10: 17 cases, 20.5% in a total of 83 cases).

### 5.4 Differential diagnosis

It mainly should be distinguished from other known viral virus of pneumonia, such as influenza viruses, parainfluenza virus, adenovirus, respiratory syncytial virus, rhinovirus, human metapneumovirus, SARSr-CoV, etc.; and also from mycoplasma pneumonia, chlamydia pneumonia, and bacterial pneumonia. In addition, it should be distinguished from non-infectious diseases, such as vasculitis, dermatomyositis, and organizing pneumonia.

### 5.5 Techniques for laboratory tests

#### 5.5.1 Hematology examination

In the early stage of the disease, the total number of leukocytes decreased or keeps normal, with decreased lymphocyte count or increased or normal monocytes. High attention should be paid on the situation where the absolute value of lymphocyte is less than 0.8 × 10^9^/L, or the numbers of CD4 and CD8 T cells are significantly decreased, which generally recommend rechecking the blood routine changes after 3 days.

#### 5.5.2 Detection of pathogens in respiratory tract

1. Flu antigens. At present, routinely detected flu antigens are A, B, and H7N-subtypes. Sampling of throat swabs is conducive to early rapid screening for flu because of the fast test, but it has a relatively high false negative rate.
2. Respiratory virus nucleic acid. The detection of respiratory virus nucleic acid is commonly used to detect the infection by other common respiratory viruses, mycoplasma and chlamydia infection, such as adenovirus, parainfluenza virus, respiratory syncytial virus, mycoplasma, chlamydia, influenza A and influenza B virus, etc.
3. 2019-nCoV nucleic acid detection. Accurate RNA detection of 2019-nCoV is with diagnostic value (*Strong recommendation*). The RNA of 2019-nCoV positive in the throat swab sampling or other respiratory tract sampling by fluorescence quantitative PCR method, especially that from multiple samples and detection kits, excluding sample quality, sample collection time, contaminatory and technical problems, is of great support for etiological diagnosis.
4. Other laboratory testing. There are other laboratory tests for the status of 2019-nCoV infection, including blood gas analysis, liver and kidney function, myocardial enzyme, myoglobin, erythrocyte sedimentation rate (ESR), C-reactive protein (CRP), Procalcitonin (PCT), lactate, D-dimer, coagulation image, urine routine test, inflammatory factors (interleukin(IL)-6, IL-10, TNF - α), 11 items of tuberculosis (TB) subgroup, complement, anti-acid staining, etc. Blood gas analysis is helpful to judge the oxygenation of moderately-illed and severe patients. Combining the increase of lactic acid, it is feasible to screen the patients with high-risk of oxygenation disorder. Some infected patients have increased liver enzymes, muscle enzyme, ESR and myoglobin. The detection of CRP and PCT is of certain value to distinguish whether there was bacterial infection in the lung. D-dimer of most severe patients was significantly increased in this epidemic, with frequent clotting disorders and microthrombotic formation in peripheral blood vessels. Detection of other inflammatory factors may help to preliminarily evaluate the immune status of patients.

#### 5.5.3 Clinical data from Zhongnan Hospital of Wuhan University

In the early stage of this disease, the total number of leukocytes in peripheral blood was normal or decreased, and the lymphocyte count decreased. In some patients, liver enzyme (transaminase), creatine kinase (CK) and myoglobin increased. CRP, ESR, and IL-6 increased, and PCT was normal in most patients. The increased D-dimer occurred in severe cases.

The data from the first 38 patients with 2019-nCoV infection who hospitalized in Zhongnan Hospital of Wuhan University were collected. Analysis revealed that the average value of white blood cells (WBC) was 5.45 (2.30–13.82) × 10^9^/L, PLT was 164.5 (47–317) × 10^9^/L, lymphocyte was 0.87 (0.24–2.27) × 10^9^/L, and monocyte was 0.38 (0.12–0.62) × 10^9^/L. The average value of ALT (alanine aminotransferase) was 37.6 (6–128) U/L and AST (aspartate aminotransferase) was 53.3 (18–169) U/L. The average value of CK was 315 (33–3051) U/L, ESR was 29.3 (8–67) mm/h, CRP was 61.8 (3–170.91) mg/L, IL-6 was 57 (3.1–134.4) pg/ml, and D-dimer was 400 (46–3330) ng/ml.

Compared with 120 healthy check-ups, the absolute value of lymphocyte (0.87 vs 2.13) × 10^9^/L, lymphocyte percentage (19.5% *vs* 33.7%), eosinophil percentage (0.13% *vs* 2.16%), and absolute value (0.0061 *vs* 0.1417) × 10^9^/L in 2019-nCoV patients were significantly reduced (*P* < 0.05). The absolute number (4.2 *vs* 3.7) × 10^9^/L and the percentage (72.0% *vs* 57.0%) increased in 2019-nCoV patients (*P* < 0.05). The percentage of monocytes increased slightly (8.1% *vs* 6.8%), while the absolute number of monocytes did not change significantly (0.38 *vs* 0.44) × 10^9^/L.

### 5.6 Other early diagnosis methods

The next generation sequencing (NGS) and electron microscope technology play a role in the early diagnosis, but their diagnostic values have been weakened by the discovery of specific nucleic acid detection technology. In addition, NGS detection can tell whether the pathogen has mutated or not.

## 6 Treatment and control

### 6.1 Principles

Suspected and confirmed cases need to be treated in designated hospitals with effective isolation and protection conditions. Suspected cases need to be treated separately in single room, confirmed cases are admitted to a same ward, and critical cases should be admitted to ICU as soon as possible.

### 6.2 Treatment plans

1. The patient should rest in bed, being monitored for vital signs (heart rate, pulse oxygen saturation, respiratory rate, blood pressure) and given supportive treatment to ensure sufficient energy intake and balance for water, electrolytes, acid-base levels and other internal environment factors *(Strong recommendation).*
2. The patient should be monitored for blood routine, CRP, PCT, organ function (liver enzyme, bilirubin, myocardial enzyme, creatinine, urea nitrogen, Urine volume, etc.), coagulation function, arterial blood gas analysis and chest imaging *(Strong recommendation).*
3. The patient should be given effective oxygen therapy, including nasal catheter, mask oxygen, high flow nasal oxygen therapy (HFNO), non-invasive ventilation (NIV) or invasive mechanical ventilation *(Strong recommendation).*

First, oxygen therapy is the choice for patients with severe respiratory infections, respiratory distress, hypoxemia or shock. The initial flow rate is 5 L/min, and the titration flow rate is to reach the target oxygen saturation (adults: SpO_2_ ≥ 90% in non-pregnant patients, SpO_2_ ≥ 92–95% in pregnant patients; children: SpO_2_ ≥ 94% in children with obstructive dyspnea, apnea, severe respiratory distress, central cyanosis, shock, coma or convulsions, and ≥ 90% in other children).

Second, respiratory support should be given to patients with hypoxic respiratory failure and acute respiratory distress syndrome. HFNO or NIV can be selected when nasal cannula or mask oxygen therapy was ineffective or the patient had hypoxic respiratory failure. However, when patients had hypercapnia (acute exacerbation of chronic obstructive pulmonary disease, cardiogenic pulmonary edema), hemodynamic instability, multiple organ failure, and abnormal mental status HFNO oxygen is not the routinely adopted measure. If respiratory failure cannot be improved or worsens continuously within a short time (1 h) after using HFNO or NIV, intubation should be performed immediately. Low tidal volume (4-8 ml/kg) and low suction pressure (platform pressure < 30cmH_2_O) are used for invasive mechanical ventilation. It is suggested that positive end-expiratory pressure (PEEP) with high positive end-expiratory pressure should be used in patients with moderate or severe acute respiratory distress syndrome, and PEEP should be titrated according to FiO_2_ to maintain SpO_2_, in order to improve alveolar atelectasis and reduce alveolar hyper-expansion and pulmonary vascular resistance at the end of inspiration. For severe patients with ARDS, it is recommended to ventilate in prone position for more than 12 h/d.

(4) Extracorporeal Membrane Oxygenation (ECMO) should be considered for the patients with refractory hypoxemia that is difficult to be corrected by protective lung ventilation. *(Strong recommendation).*

### 6.3 Drug treatment

#### 6.3.1 Antiviral treatment

1. At present, there is no evidence from RCT to support specific drug treatment against the new coronavirus in suspected or confirmed cases.
2. The α-interferon atomization inhalation can be considered (5 million U per time for adults in sterile injection water, twice a day) *(Weak recommendation)*; lopinavir/ritonavir orally, 2 capsules each time, twice a day, can be also considered *(Weak recommendation)*.

Low-level evidences included retrospective cohort, historically controlled studies, case reports, and case series revealed that lopinavir/ritonavir alone or in combination with antivirals produced certain benefits in the treatment of SARS and MERS, such as reducing the incidence or mortality of ARDS [26–29]. A recently systematic review showed that lopinavir/ritonavir’s anti-coronavirus effect was mainly seen in its early application, for reducing patient mortality and reduced glucocorticoid consumption. However, if the early treatment window is missed, there will be no significant effect in their late application [30]. Real-world study stills need to further explore the clinical effects of its early use in 2019-nCoV infected pneumonia.

The effectiveness of the combined use of antivirals is still controversial [31–34].

#### 6.3.2 Antibiotic therapy

1. Principles. Avoid blind or inappropriate use of antibacterial drugs, especially the combination of broad-spectrum antibacterial drugs. Enhancement of bacteriological surveillance should be performed and promptly given appropriate antibacterial drugs when it occurs secondary bacterial infection.
2. According to the clinical manifestations of patients, if the accompanying bacterial infection cannot be ruled out, mild patients can take antibacterial drugs against community-acquired pneumonia, such as amoxicillin, azithromycin, or fluoroquinolones; empirical antibacterial treatment in severe patients should cover all possible pathogens, deescalating therapy until the pathogenic bacteria are clarified.

#### 6.3.3 Corticosteroid therapy

The use of corticosteroids for severe ARDS is controversial; therefore, systemic use of glucocorticoids needs to be cautious. Methylprednisolone can be used as appropriate for patients with rapid disease progression or severe illness. According to the severity of the disease, 40 to 80 mg of methylprednisolone per day can be considered, and the total daily dose should not exceed 2 mg/kg *(Weak recommendation)*.

SARS management related researches showed that timely use of non-invasive continuous positive airway pressure and corticosteroids is an effective strategy for increased lung shadows and increased dyspnea. Appropriate use of glucocorticoids is able to significantly improve the clinical symptoms of patients with SARS, reduce the degree of disease progression, and accelerate the absorption of lung lesions; but it cannot shorten the length of hospital stay [35, 36]. Be cautious that hormone therapy has some incidence of adverse reactions [37].

#### 6.3.4 Other medications

1. Symptomatic treatment of fever. When the temperature is higher than 38.5 ℃, ibuprofen can be used for antipyretic (oral, 0.2 g per time, it can be used every 4–6 h in continuous fever, but no more than 4 times in 24 h), and the temperature below 38 ℃ is acceptable. Much lower body temperature is not conducive to antiviral treatment.
2. Nutrition support treatment. Inpatients are screened for nutrition risk based on the NRS2002 score when they are admitted to the hospital. The recommended plan for patients with different nutrition risk scores are as follows:
  First, if the total score is < 3 points, it is recommended to eat protein-rich foods (such as eggs, fish, lean meat, dairy products) and carbohydrate-containing diets. The supposed ideal energy intake is 25–30 kcal / (kg∙d) and the protein mass are 1.5 g / (kg∙d).
  Second, if the total score is ≥3 points, the patient should be given nutritional support as early as possible. It is recommended to increase protein intake by oral nutrition supplement, 2–3 times/day (≥ 18 g protein/time). In order to reach the mount of 18 g protein/time, protein powder can be added on the basis of standard whole protein preparations. Enteral nutrition tube should to be placed when the patient cannot intake supplemental nutrition by oral routine.
3. Reduce the incidence of stress ulcers and gastrointestinal bleeding. Use H_2_ receptor antagonists or proton pump inhibitors in patients with gastrointestinal bleeding risk factors. The risk factors for gastrointestinal bleeding include mechanical ventilation ≥48 h, coagulation dysfunction, renal replacement therapy, liver disease, various complications, and a higher score of organ failure.
4. Reduce the secretion of lung glands and improve the respiratory function. For patients with dyspnea, cough, wheeze, and respiratory distress syndrome due to the increased respiratory gland secretion, it is recommended to use selective (M1, M3) receptor anticholinergic drugs to reduce the secretion, relax the smooth muscle in airway, relieve airway spasm and improve the pulmonary ventilation.
5. Reduce the incidence of venous embolism. Evaluate the risk of venous embolism in patients and use low-molecular-weight heparin or heparin in high-risk patients without contraindications.

### 6.4 Traditional Chinese medicine treatment

#### 6.4.1 Guiding principles

Treat the patient based on syndrome differentiation individually. Prevention before illness is better than treatment after getting diseased.

#### 6.4.2 Prevention

1. Community. Implement relevant national regulations and take great effort to keep away from contaminated materials, disinfect the environment, and improve the healthcare management.
2. Individual. It is recommended to take food in proper amount and balanced nutrition, have regular daily life and physical activities, and avoid overloaded work.
3. Psychology. Develop proper interests and career in a mutual promoting manner.
4. Drug. Including:
  i Fumigation with moxa in the room, 1-5 g/m^2^ for 30 min per day.
  ii Wearing perfumed Chinese herb bags (clove, fineleaf schizonepeta herb, *Perilla frutescens*, atractylodes lancea, cinnamon, biond magnolia flower, asarum sieboldii, and *Elettaria cardamomum*, 2 g for each, crushed into powder and put it into bags for external use, change a new one every 10 days).
  iii Prescription of Chinese herbs for feet bath (vulgaris 10 g, carthamus 10 g, and dried ginger 6 g) Soaking the herbs in boiling water and bath the feet into the medical liquid when the temperature is suitable. Soak feet for about 20 min.
  iv Prescription of Chinese herbs for prophylaxis: *Astragalus mongholicus* 12 g, roasted rhizoma atractylodis macrocephalae 10 g, saposhnikovia divaricata 10 g, *Cyrtomium fortunei* 10 g, honeysuckle 10 g, dried tangerine or orange peel 6 g, eupatorium 10 g, and licorice 10 g. Taking the medicine above yielded decoction once a day for adults, and for 5 days as a treatment course. If for children, cutting the dose to half.
  v Medical tea: perilla leaf 6 g, agastache leaf 6 g, dried tangerine or orange peel 9 g, stewed amomum tsao-ko 6 g, and 3 slices of ginger. Soak the herbs in hot water and drink the water just like enjoying the tea.
  vi Chinese patent medicine: Huoxiang Zhengqi capsule or Huoxiang Zhengqi Shui (in half dose).

#### 6.4.3 Treatment [12]

##### In medical observation period

There are two clinical symptoms in this period, including:

1. Clinical symptoms 1: hypodynamia accompanied by gastrointestinal upset. And the recommended Chinese patent medicine is the Huoxiang Zhengqi capsules (ball, liquid, or oral liquid).
2. Clinical symptoms 2: hypodynamia and fever. And the recommended Chinese patent medicines is the Jinhua Qinggan granules, Lianhua Qingwen capsules (granules), Shufeng Jiedu capsules (granules), or Fangfeng Tongsheng pills (granules).

##### Clinical treatment period

This period involving 7 stages, including:

1. Early-stage, characterized as exterior syndrome of cold-dampness. In this stage, the clinical manifestations presents as follow: aversion to cold without sweating, headache and generalized heaviness, limb pain, glomus and fullness in the chest and diaphragm, thirst with no desire to drink, ungratifying loose stool, yellow urine, frequent micturition and yellow urine. The therapeutic logic is to dissipate cold and eliminate dampness. And the recommended prescription is the Huoxiang Zhengqi powder (Yin dampness injuring superficies case from the *National Famous Traditional Chinese Medical Doctor Medical Cases*); which comprises of perilla leaf 10 g, atractylodes lancea 15 g, radix angelicae dahuricae 10 g, dried tangerine or orange peel 10 g, notopterygium root 10 g, agastache rugosus 10 g (end addition), mangnolia officinalis 10 g, saposhnikovia divaricata 10 g, poria peel 15 g, and *Tetrapanax papyriferus* 10 g above yielded decoction. In addition, the recommended Chinese patent medicine is Huoxiang Zhengqi capsules or Huoxiang Zhengqi Shui.
2. Early-stage, characterized as cold-dampness obstructing lung. In this stage, the clinical manifestations presents as follow: aversion to cold with or without fever, dry cough, dry throat, fatigue and hypodynamia, oppression in chest, epigastric fullness, or nausea, loose stool. The tongue is pale or reddish, the tongue fur is slimy white, and soggy pulse. Hence, the therapeutic logic is to dissipate cold and resolve obstruction. And the recommended prescriptions comprises of atractylodes lancea 15 g, dried tangerine or orange peel 10 g, mangnolia officinalis 10 g, agastache rugosus 10 g (end addition), amomum tsao-ko 6 g, ephedra herb 6 g, notopterygium root 10 g, ginger 10 g, areca-nut 10 g (end addition), periostracum cicada 10 g, bombyx batryticatus 10 g, and rhizoma curcumae longae 10 g above yielded decoction.
3. Middle-stage, characterized as epidemic toxin blocking the lung. In this stage, its clinical manifestations includes persistent fever or alternating cold and heat, cough with less phlegm, or yellow phlegm, abdominal distension and constipation; oppression in chest with anhelation, cough with wheezes, panting on exertion; or red tongue, slimy yellow fur or yellow dry fur, slippery and rapid pulse. Therefore, the therapeutic logic is clearing heat and detoxicating. And the recommended prescription comprises of almond 10 g, gypsum 30 g (predecoction), trichosanthes kirilowii 30 g, rhubarb 6 g (end addition), ephedra with honey fried 6 g, semen lepidii 10 g, peach kernel 10 g, amomum tsao-ko 6 g, areca-nut 10 g, and atractylodes lancea 10 g above yielded decoction.
  In addition, the recommended Chinese patent medicine is Xiyanping injection or Xuebijing injection.
4. Severe stage, characterized as heat toxin generating stasis. In this stage, the clinical manifestations is known as high fever, oppression in chest with anhelation, purple-black facial complexion, lips dark and swollen, obnubilation, crimson tongue, yellow dry fur, surging and fine rapid stringlike pulse. Thus, its therapeutic logic is detoxicating and dispersing blood stasis.
  The recommended prescription is three Yellows and Gypsum decoction, Shang Jiang Powder, and Toxin-Resolving Blood-quickening decoction. Its composition comprises of ephedra with honey fried 10 g, almond 10 g, gypsum 20-30 g, periostracum cicada 10 g, bombyx batryticatus 10 g, rhizoma curcumae longae 10 g, rhubarb stir-fried with wine 10 g, scutellaria baicalensis 10 g, coptis chinensis 5 g, phillyrin 15 g, angelica sinensis 10 g, peach kernel 10 g, radix paeoniae rubra 15 g, and rhizome of rehmannia 15 g above yielded decoction.
  The recommended Chinese patent medicines is the Xiyanping injection, Xuebijing injection, Qingkailing injection, or Angong Niuhuang pills.
5. Severe-stage, characterized as inner blocking causing collapse. In this stage, the clinical manifestations include dyspnea, panting on exertion or need assisted ventilation, accompanied by coma, and agitation, cold limbs with cold sweating, dark purple tongue, thick or dry thick tongue fur, floating and rootless pulse. The thrapeutic logic is rescuing from collapse by restoring Yang. Hence, the recommended prescription comprises of ginseng 15 g, aconitine 10 g (predecoction), and *Cornus officinalis* 15 g above yielded decoction, and both taken with fluid Suhexiang pills or Angong Niuhuang pills.
  The recommended Chinese patent medicines is Xuebijing injection, Shenfu injection, or Shengmai injection.
6. Recovery-stage, presents as lung and spleen Qi deficiency. Its clinical manifestations include shortness of breath, fatigue and hypodynamia, anorexia, nausea and vomiting, glomus and fullness, weak stools, ungratifying loose stool, pale tender-soft enlarged tongue, slimy white tongue fur. Therefore, therapeutic logic is to supplement the spleen and lung.
  The recommended prescription comprises of rhizoma pinellinae praeparata 9 g, dried tangerine or orange peel 10 g, *Codonopsis pilosula* 15 g, radix astragali preparata 30 g, poria cocos 15 g, agastache rugosus 10 g, and fructus amomi 6 g (end addition) above yielded decoction. In addition, the recommended Chinese patent medicines is pill of costus and amomum with six noble ingredients.
7. Recovery-stage, characterized as deficiency of Qi and Yin. The clinical manifestations of this stage is generalized heat with sweating, chest heat vexation, Qi counterflow with retching and vomiting, shortness of breath and lassitude of essence-spirit, red tongue and thin tongue fur, vacuous pulse. Hence, the therapeutic logics is boost Qi and nourish Yin.
  The recommended prescription is Zhuye Shigao decoction with cogongrass rhizome and rhizoma phragmitis; and the composition of this prescription includes bamboo leaf 15 g, gypsum 15 g (predecoction), *Codonopsis pilosula* 15 g, radix ophiopogonis 10 g, pinellia ternate 9 g, cogongrass rhizome 15-30 g, rhizoma phragmitis 20 g, licorice 10 g, and polished round-grained rice 30 g above yielded decoction.
  The recommended Chinese patent medicine: Shengmaiyin.

### 6.5 Treatment of severe patients

#### 6.5.1 Hypoxemic respiratory failure and ARDS treatments

Treatment principle: treat the patients to improve the symptoms and underlying diseases, actively prevent potential complications and secondary infection; provide timely measures to support organ function.

1. Hypoxic respiratory failure and severe ARDS. Give oxygen therapy immediately to patients with ARDS, and closely monitor them for signs of clinical deterioration, such as rapidly progressive respiratory failure. Consider severe hypoxemic respiratory failure when standard oxygen therapy fails. When patients have increased frequency of breathing (> 30 times/min) and hypoxemia (SpO_2_ < 90% or PaO_2_ < 60 mmHg) even with oxygen delivered via a face mask and reservoir bag (gas flow of 10~15 L/min, FiO_2_ 0.60–0.95), it may be considered as hypoxic respiratory failure.
  ARDS is a status of severe acute hypoxic respiratory failure caused by increased pulmonary capillary permeability and alveolar epithelial cell damage. It can be divided into mild, moderate and severe conditions according to the Berlin definition [38] (Table 6).

**Table 6:** The Berlin definition for acute respiratory distress syndrome

Item	Mild	Moderate	Severe
Onset time	Respiratory symptoms developed/aggravated within 1 week after clinically known damage		
Hypoxemia	PaO_2_/FiO_2_ 201–300 mmHg, PEEP or CPAP ≥5 cmH_2_O	PaO_2_/FiO_2_ 101–200 mmHg, PEEP≥5 cmH_2_O	PaO_2_/FiO_2_ ≤ 100 mmHg, PEEP≥10 cmH_2_O
Causes of pulmonary edema	Respiratory failure cannot be completely explained by heart failure or fluid overload. Objective assessment (such as echocardiography) is needed to eliminate the possibility of hydrostatic pulmonary edema if other risk factor is absent.		
Abnormality in imaging	Decreased transparence of two lungs cannot be completely explained by pleural effusion, atelectasis or nodules.		

*PEEP* positive end-expiratory pressure, *CPAP* continuous positive airway pressure
2. HFNO. Under the support of standard oxygen therapy, to maintain SpO_2_ above 93% stills hard, and the breathing rate increases rapidly, then HFNO should be considered. HFNO can deliver 60 L/min of gas flow and FiO_2_ up to 1.0. Generally, gas flow is initially set as 30–40 L/min and oxygen concentration 50%–60%, which is well tolerated and coordinated. Then settings can be adjusted according to the oxygenation status of patients. Compared with standard oxygen therapy, HFNO is able to reduce the chance of tracheal intubation. Patients with hypercapnia (like exacerbation of obstructive lung disease, cardiogenic pulmonary edema), hemodynamic instability, multi-organ failure, or abnormal mental status should not be given HFNO. HFNO may be safe in patients with mild-moderate and non-worsening hypercapnia. However, if the respiratory distress still exists or even worsens dramatically under HFNO (FiO_2_ > 70%, gas flow > 50 L/min for 1 hour), the respiratory supporting strategy should be changed.
3. NIV. NIV provides a certain positive pressure ventilation effect through the positive pressure formed by the closed mask. HFNO combined with intermittent short-term NIV (1–2 h) support may be useful to reduce respiratory power consumption and improve oxygenation. But NIV guidelines recommend the use of respiratory support therapy in hypoxemic respiratory failure or pandemic viral illness. Limited data showed a high failure rate of NIV in MERS patients. Invasive mechanical ventilation should be considered in case the ARDS still exists and even acutely deteriorates in NIV process (about 1 h). Patients with hemodynamic instability, multiple organ failure, or abnormal mental status should not receive NIV.
4. Invasive mechanical ventilation. Under the support of HFNO (the demand for FiO_2_ > 70% and gas flow > 50 L/min) or NIV, ARDS still exists and even acutely deteriorates, invasive mechanical ventilation should be implemented as soon as possible.
  Endotracheal intubation should be carried out by trained and experienced provider using airborne precautions, since endotracheal intubation is an operation that may produce a large number of contagious aerosols.
  The strategy of protective lung ventilation should be implemented in invasive mechanical ventilation: lower tidal volume (4–6 ml/kg), lower plateau pressure (< 30 cmH_2_O), and appropriate PEEP. For patients with moderate-severe ARDS (PaO_2_/FiO_2_ < 150), it is recommended to use higher PEEP, apply prone ventilation for more than 12 h per day and adopt deep sedation and analgesia muscle relaxation strategy within the first 48 h of mechanical ventilation. For patients with severe acute hypoxic respiratory failure, we should pay attention to and prevent ventilator-associated lung injury after mechanical ventilation.
5. Extracorporeal Life Support (ECLS). In the process of invasive mechanical ventilation when the patient is still in the state of hypoxia, combined with increased partial pressure of carbon dioxide (excluding ventilation dysfunction, PaCO_2_ > 60 mmHg), especially after muscle relaxation and prone ventilation, it is necessary to consider to implement ECLS. However, it is suggested that ECLS treatment should only be carried out under the condition that the professional center is with access to expertise. Currently the ECLS in ICU includes VV-ECMO (blood is pumped from femoral vein, and returns to right atrium through internal jugular vein after oxygenation through membrane oxygenator) and VA-ECMO (blood is pumped from femoral vein and directly enters aortic system through femoral artery after oxygenation through membrane oxygenator). For patients with severe refractory hypoxemia, neuromuscular blockade can improve oxygen supply, especially if there is still evidence of ventilator-patient dyssynchrony after the use of sedatives. However, neuromuscular blockade through continuous infusion should not be routinely used in patients with moderate-severe ARDS; Where available, ECMO in conjunction with low tidal-volume mechanical ventilation can be considered in the treatment of patients with severe refractory hypoxemia in whom standard therapy are failing; Routine use of high-frequency oscillatory ventilation (HFOV) in patients with moderate-severe ARDS is not beneficial, but may be harmful. However, HFOV may still be regarded as a rescue therapy for patients with severe ARDS and refractory hypoxemia. ECMO can be used in some severe ARDS patients (lung injury score > 3 or pH < 7.2 due to uncompensated hypercapnia), but it is not recommended for all ARDS patients. It can be considered to use extracorporeal carbon dioxide removal for ARDS patients, if there is more supportive research evidence in the future.

Conservative fluid management can be adopted for ARDS patients without tissue hypoperfusion. Use vasoactive drugs to improve microcirculation. Empirical antibiotics targeting the suspected potential infection should be used as soon as possible, blind or improper combination of broad-spectrum antibiotics should be avoided. Unless for special reasons, the routine use of corticosteroids should be avoided. Glucocorticoids can be used in a short time (3–5 days) according to the degree of dyspnea and the progress of chest imaging if appropriate and the recommended dose is not more than the equivalent to 1-2 mg/kg methylprednisone per day. Provide intensive standard supportive care to the critically ill patients, including prevention of deep vein thrombosis and stress-induced gastrointestinal bleeding, blood glucose control and so on. Enteral nutrition can be provided. Supplemental nutrition with omega-3 fatty acids and antioxidants is not recommended. Inhaled or intravenous beta-adrenergic agonists are not recommended to promote alveolar fluid clearance and resolution of pulmonary edema.

#### 6.5.2 Treatment of septic shock

1. Recognize the septic shock. When infection is suspected or confirmed, and on the basis of full fluid resuscitation, vasoconstrictor drugs are still needed to maintain mean arterial pressure (MAP) ≥65 mmHg with lactate ≥2 mmol/L, the existence of septic shock should be considered. If lactate cannot be monitored for some reasons, the following three manifestations (changes in mental state, oliguria, poor peripheral perfusion and prolonged capillary filling time) should be considered as signs of a combination of infection and hypoperfusion.
2. In resuscitation from septic shock in adults, at least 30 ml/kg of isotonic crystalloid was considered for adults in the first 3 h. In resuscitation from septic shock in children, give 20 ml/kg as a rapid bolus and up to 40–60 ml/kg in first aid.
3. Isosmotic crystal solution is recommended for resuscitation. Do not use hypotonic crystalloids, starches, or gelatins for resuscitation in the first hour. Albumin may be considered as a resuscitation fluid, but this recommendation was based on low- quality evidence under certain conditions.
4. Administer vasoconstrictor is suggested when shock persists after fluid resuscitation, noradrenaline as the first choice. The initial blood pressure target is MAP ≥65 mmHg in adults and age-appropriate targets in children.
5. If it is not possible to place a central venous catheter, vasopressors can be infused through the peripheral vein through large vein and signs of extravasation and local tissue necrosis should be closely monitored.
6. If extravasation occurs, stop infusion. Vasopressors can also be administered via intraosseous needles.

### 6.6 Condition evaluation and treatment effect evaluation

#### 6.6.1 Criteria to withdraw ECLS

1. Remove VV-ECMO. The oxygen concentration of the ECMO air-oxygen mixer has dropped to 21%, the air flow rate has dropped to 0, and the ventilator is not strong enough. Lasting for 2–3 h, the respiratory rate is within 25 breaths/min, SpO_2_ > 92%, PaCO_2_ is normal, and withdrawal from VV-ECMO may be considered.
2. Remove VA-ECMO. The blood flow rate is reduced to the rate of (0.2 to 0.5 L / min) every 5 to 6 h from 3 L/min, and the hemodynamic condition is stable. The blood flow rate is reduced to 1.5 L/min within 24 h. If there is a bridging tube, the arteriovenous end can be connected with a bridging tube to form an ECMO circuit for self-circulation, so that the body’s hemodynamics is driven by the heart. If hemodynamics is stable for at least 6 h, consider removing the machine.

#### 6.6.2 Criteria for removing invasive breathing

When the patient is well aware, cough reflexes are obvious when sucking the sputum, the hemodynamics is stable, and the ventilator parameters are close to offline parameters, the spontaneous breathing test (SBT) is performed. After the SBT is passed, invasive breathing can be considered to remove the endotracheal tube.

#### 6.6.3 Standards of transfer out of ICU

Patients do not need advanced respiratory support (HFNO, NIV, MV, ECLS, etc.); stable hemodynamics and tissue perfusion; no significant impairment of organ function; and no need for organ support treatment (CRRT, artificial liver, etc.). Consider transferring the patient out of ICU procedure.

### 6.7 Discharge standards

The body temperature returned to normal for more than 3 days; respiratory symptoms improved significantly; inflammation of the lungs showed obvious signs of absorption; and respiratory nucleic acid was negative for two consecutive times (one-day sampling time interval at least); and the patient can be released from isolation.

## 7 Prevent and control nosocomial infection

### 7.1 Restriction and isolation guidelines for patient/ suspected patients

See Table 7. *(Strong recommendation)*.

**Table 7:** Restriction and isolation guidelines checklist for patients/suspected cases *(Strong recommendation)*

Category	Tactics	Precautions in practice
Environmental requirements	1.There should be clean areas, potentially contaminated areas, contaminated areas, contaminated channels and clean channels	1.1 clearly arrange and mark the 3 areas and transport materials or move from clean area to contaminated area. Retrograde is not allowed.
		1.2 Each area should be physically partitioned and clearly marked
	2. Isolation in single (priority strategy) Collective isolation for the confirmed patients, collective isolation for the suspected cases (alternative strategy)	2.1 < 4 persons per isolation ward, bed spacing ≥1.1 m
		2.2 Equipped with separate toilet
		2.3 Equipped with hand-cleaning and disinfection apparatus
		2.4 Minimize the unnecessary items (e.g. remove the curtains, etc.)
	3. Ensure that the environment and articles are clean and disinfected	3.1 Follow the Disinfection Guidelines checklist
		3.2 Exclusive use of articles in isolation areas
	4. Proper medical waste management	4.1 The medical waste should be put in sealed double-layer yellow medical waste bags for regulated disposal procedure.
Requirements to the patient/suspected Patient	5. Restrict the range of patient/suspected patient for their activities.	5.1 No escort or minimize the number of escorts.
		5.2 Clear route for patient transport (get in or out through contaminated channels)
		5.3 Patients going out should wear N95 masks or surgical masks
		5.4 Follow the disinfection guidelines after being discharged from hospital.
Requirements to the medical staff request	6. Medical personnel enter the isolation area with proper self-protection through designated channels.	6.1 Medical staff should perform the personal protection practice under the Personal Protection Guidelines in Table 8

### 7.2 Personal protection guidelines

According to the principles of standard prevention and tertiary protection, all personnel entering various zones should be evaluated using individual inventory tables according to the exposure risk level. Chose personal protective equipment of various levels is necessary. Personal protective equipment should be worn strictly in accordance with the instructions and only used for one time (Table 8, *Strong recommendation*).

**Table 8:** Personal protection guidelines checklist *(Strong recommendation)*

Item	Exposure intensity of infection risk^a^	Protective measurement								
		Round hat	N95 mask	Coverall	Eye protector/Protective panel	Latex gloves	Barrier gown	Protective clothing	Shoe cover/Bootstrap	Comprehensive respiratory apparatus
Recommendations as per work area										
Pre-examination triage	Low	✓	✓	✓		✓	✓			
General out-patient service	Low	✓	✓	✓		✓				
General ward	Medium	✓	✓	✓	✓	✓	✓			
	High	✓	✓	✓	✓	✓	✓	✓		
Fever clinic	Medium	✓	✓	✓	✓	✓	✓	✓	✓	
	High	✓	✓	✓	✓	✓	✓	✓	✓	✓
Isolation room (Area)	Medium	✓	✓	✓	✓	✓	✓	✓	✓	
	High	✓	✓	✓	✓	✓	✓	✓	✓	✓
Department of infectious diseases	Medium	✓	✓	✓	✓	✓	✓	✓	✓	
	High	✓	✓	✓	✓	✓	✓	✓	✓	✓
Recommendations as per personnel										
Medical staff in the isolation area	High	✓	✓	✓	✓	✓	✓	✓	✓	✓
	Medium	✓	✓	✓	✓	✓	✓	✓	✓	
Staff in pre-examination triage	Medium	✓	✓	✓	✓	✓	✓	✓	✓	
Medical staff in Out-patient Department	Medium	✓	✓	✓	✓	✓	✓	✓	✓	
Medical staff in the observing ward	High	✓	✓	✓	✓	✓	✓	✓	✓	✓
	Medium	✓	✓	✓	✓	✓	✓	✓	✓	
Assisting staff	Medium	✓	✓	✓	✓	✓	✓			
Administrative and supporting staff	Low	✓	✓	✓	✓	✓	✓			

^a^Low risk, general contact with patients or exposure to contaminated environment, such as escorting the patients during diagnosis, triage, palpation, consultation, etc

Medium risk, direct contact with body fluid, mucosa or incomplete skin, such as oral examination, puncture, oral care, surgery, etc

High risk, there is a risk of spatter of secretions or contaminants onto the body and face of medical staff, such as oral diagnosis, endotracheal intubation, etc

## 8 Disease nursing

### 8.1 Nursing of isolated patients at home

The patient’s home isolation scheme is shown in Table 5.

Patients should monitor their body temperature and illness at home. If your body temperature continues to be higher than 38 ℃, or your breath is getting worse, you should seek medical treatment timely.

In addition to taking protective measures, the home caregivers also should monitor their body temperature closely.

### 8.2 Nursing the patients

#### 8.2.1 Nursing of oxygen therapy

Mild patients generally use a nasal catheter and a mask for oxygen. Adjust the oxygen flow as appropriate according to the patient’s condition and doctor’s instruction, and closely monitor the patient’s breathing and blood oxygen saturation. If oxygen therapy fails to reach the expected effect, the nurse should analyze the cause comprehensively and be vigilant to notify the doctor.

#### 8.2.2 Nursing of medication

Mild patients generally use antiviral drugs, antibacterial drugs (when bacterial infection exists), and symptomatic treatment. The doctor’s advice should be followed accurately and timely. The adverse reactions of oseltamivir mainly include nausea, vomiting, diarrhea, abdominal pain and bronchitis, cough, etc. The adverse reactions of interferon are mainly flu-like symptoms such as fever, fatigue, myalgia, and headache, followed by mild suppression of bone marrow. Attention should be paid to identify the change of clinical manifestations or adverse drug reactions.

#### 8.2.3 Nutritional support

According to the patients’ condition, provide high-protein, high-vitamin, carbohydrate-containing diets (e.g. eggs, fish, lean meat, milk, etc.) for enough nutrition to improve physical condition.

#### 8.2.4 Psychological nursing

Take good care of the patient and respond to the patient’s question timely. Positively encourage patients to reduce their anxiety and fear.

### 8.3 Nursing of critically illed patients

#### 8.3.1 Condition monitoring

Dynamically monitor patients’ vital signs, water-electrolytes balance, acid-base balance, and functions of various organs, monitor patients’ infection indicators, and determine the occurrence of complications such as acute respiratory distress syndrome, septic shock, stress ulcers, and deep vein thrombosis.

#### 8.3.2 Sequential oxygen care

The critically illed patients mainly use oxygen therapy such as HFNO, NIV and invasive mechanical ventilation. When using various oxygen treatments in a sequential manner, the airway and breathing circuit need to be kept open, and the effect of oxygen treatment needs to be monitored dynamically. At the same time, skincare products need to be used reasonably to avoid damage to the nose, face and lips by pressure. When using a high-flow nasal catheter to inhale oxygen, the oxygen flow and temperature and humidity should be adjusted appropriately. When using non-invasive mechanical ventilation, patient should receive relevant health education. Patients are instructed to inhale through the nose. The pressure is set from low to high and gradually reaches the target value. The human-machine coordination is maximized. The patient’s consciousness and respiratory function are closely observed. Patients with artificial airway established should use a closed suction tube to reduce virus spread. Nurses should wear goggles or a face shield to avoid occupational exposure.

#### 8.3.3 Special treatment nursing

If the patient develops moderate to severe ARDS, invasive mechanical ventilation combined with a prone position need to be adopted. Standard operating procedure for prone position needs to be followed. At the same time, be cautious to prevent pressure ulcers, falling bed, tube slippage, and eye damage by pressure and other complications. Patients treated with ECMO should be monitored for the performance of the oxygenator. If the oxygenator changes its color to darker, indicating the possibility of coagulation, the doctor should be notified to adjust the heparin dose as necessary. The oxygenator should be replaced if necessary. The coagulation function need to be monitored dynamically, including the whole set of coagulation and DIC (disseminated intravascular coagulation), and the time of activating partial thromboplastin, etc., the patient should be closely observed for signs of bleeding, such as bruising on the skin and mucous membranes, bleeding in the nasal cavity, oral cavity, bloody sputum, hematuria, blood in the stool, swelling of the abdomen, moving dullness, and the size of bilateral pupils. Make sure that the ECMO pipelines are tightly connected and firmly fixed to prevent air embolism and pipeline slippage.

#### 8.3.4 Infection prevention

Perform oral care and skin care, assist the patient to use toilet, and take eyes on the indwelling tubes. Rules and regulations for aseptic operation and isolation should be strictly followed to prevent ventilator-related pneumonia, catheter-related sepsis, urinary catheter related urinary tract infections and other secondary infections.

#### 8.3.5 Nutrition support

Dynamically assess the patients’ nutritional risks and timely nutritional support can be given if needed. For the patients who can eat, the diet rich in protein and carbohydrates is recommended. Those patients who cannot eat but are compatible with enteral nutrition should be given enteral nutrition as soon as possible. For the patients incompatible with enteral nutrition, parenteral nutrition should be given timely to meet energy requirement.

#### 8.3.6 Psychological nursing

Psychological and humanistic care should be performed in high priority especially for the awake patients. Psychological techniques like mindfulness - based stress reduction can be adopted to relieve the patients’ anxiety and panic by building up their optimistic confidence in overcoming the disease.

## 9 Limitations of this guideline

Our guideline has three major limitations: Firstly, time is so limited that we cannot fully consider all clinical issues for this emergency disease. Secondly, many evidences came from data search is indirect. Thirdly, because some recommendations are based on the evidence from existing guidelines and experts’ experience, there are situations where strong recommendations were produced on the base of low-quality evidence or very low-quality evidence, so high-quality evidence, when they appear, is likely to change current recommendations.

## 10 Supplementary information

**Additional file 1.** A successful treatment case of the severe 2019-nCoV infected pneumonia patient.

**Additional file 2.** Experience and lessons in hospital rescue for 2019-nCoV infections.

## Data Availability

The data and materials used during the current review are all available in this review.

## Abbreviations

2019-nCoV: 2019 novel coronavirus
ALT: Alanine aminotransferase
ARDS: Acute respiratory distress syndrome
AST: Aspartate aminotransferase
CCEBTCM: China Center for Evidence Based Traditional Chinese Medicine
CDC: Centers for Disease Control and Prevention
CK: Creatine kinase
CPAM: China International Exchange and Promotive Association for Medical and Health Care
CPAP: Continuous positive airway pressure
CRP: C-reactive protein
CRRT: Continuous renal replacement therapies
DIC: Disseminated intravascular coagulation
ECLS: Extracorporeal life support
ECMO: Extracorporeal membrane oxygenation
ESR: Erythrocyte sedimentation rate
GRADE: Grading of Recommendations Assessment, Development and Evaluation
HFNO: High flow nasal oxygen therapy
HFOV: High-frequency oscillatory ventilation
HRCT: High-resolution computed tomography
ICU: Intensive Care Unit
IL: Interleukin
MAP: Mean arterial pressure
MERS: Middle East respiratory syndrome
NGS: Next generation sequencing
NICE: National Institute for Health and Clinical Excellence
NIV: Non-invasive ventilation
PCT: Procalcitonin
PEEP: Positive end-expiratory pressure
PLT: Platelet
RCTs: Randomized controlled trials
SARS: Severe acute respiratory syndrome
SBT: Spontaneous breathing test
TB: tuberculosis
TNF: Tumor Necrosis Factor
WBC: White blood cells
WHO: World Health Organization
