# Supplementary material for: A rapid advice guideline for the diagnosis and treatment of 2019 novel coronavirus (2019-nCoV) infected pneumonia (standard version)
Source: Mil Med Res. 2020 Feb 6;7:4. doi: 10.1186/s40779-020-0233-6 (PMC7003341; doi:10.1186/s40779-020-0233-6)
Supplement: Supplementary file 1 — Additional file 1. A successful treatment case of the severe 2019-nCoV infected pneumonia patient. [file 40779_2020_233_MOESM1_ESM.doc]

# Additional file 1: A successful treatment case of the severe 2019-nCoV infected pneumonia patient

# 1 Treatment process

## 1.1 Patient admission

A 52-year-old male presented with a history of hypertension was admitted. He had been engaged in seafood sales in the vegetable market in Huanggang city, Hubei province; but he had no history of exposure to the Huanan seafood market since December 1, 2019. Three days ago, after returning home from the vegetable market he worked, he experienced chills, fever (a maximum of 39.0°C) and dry cough. After 3 days’ treatment in the local hospital, the lung CT showed bilateral ground-glass changes, and the patient presented dyspnea and discontinuous speech. The patient’s pulse oxygen saturation was maintained at 92%-94% using oxygen masks at an oxygen flow of 8-10L. Blood gas analysis showed SpO_2_ of 66% with PCO_2_ of 43 mmHg.

In the local hospital he was considered as severe pneumonia and type I respiratory failure. When the patient was transferred to Zhongnan Hospital of Wuhan University, the blood lymphocytes decreased significantly, and CT imaging on chest showed multiple strip-like and fine grid-like ground-glass opacities in both lungs. After multidisciplinary consultation, he was admitted for viral pneumonia. After admission, the nucleic acid amplification tests for 2019-nCoV was positive. Then, supportive treatment such as antiviral and hormone therapy was given. On the evening of 5 January 2020, he was admitted to the emergency isolation ward for fever for 3 days

## 1.2 Overview of diagnosis and treatment in our hospital

After admission, he received antiviral therapy and conventional oxygen therapy for respiratory support, but the disease was progressing. At the 3rd day after admission, the patient developed dyspnoea and wheezing, and the blood oxygen saturation lowered to 65%, and 40 breaths/min of respiratory rate. There was no improvement by non-invasive ventilation (NIV) for 1 hour, with pulse oxygen saturation maintained at 80%~85%, and blood gas analysis showed SpO_2_ of 56% with PCO_2_ of 61 mmHg. Later tracheal intubation with ventilator-assisted ventilation was performed. Re-examination of CT (Figure S1) showed significant increase in bilateral plaques and appearance of consolidation in majority of lung. Re-examination CT scan of prone position ventilation also showed obvious progress. Then tracheal intubation with invasive mechanical ventilation and lung protective ventilation strategy combined with prone position ventilation were still ineffective, occurring accumulation of carbon dioxide (PaCO_2_75mmHg).

Immediately, the diagnosis and treatment team performed extracorporeal membrane oxygenation (ECMO) on-board indication assessment and survival rate RESP assessment. The danger rating is grade III and the survival rate is about 57%. Quickly communicating with family members, the mobile ECMO supporting team center for critical illed patients in Zhongnan Hospital of Wuhan University successfully operated the machine within 20 minutes.

During VV-ECMO (Figure S2), the ECMO management team conventionally monitoring anticoagulation, oxygen flow, blood flow, and blood oxygen (before and after the membrane), then gradually reduced the supporting oxygen concentration. Daily routine chest radiographs (CR) are performed. After 5 days of VV-ECMO, the ECMO oxygen flow was turned off, and the patient was observed for 6 hours to evaluate the indications for withdrawal syndromes. The patient’s oxygenation was maintained normally, and then extubation and mechanical ventilation withdrawal were applied. The patient was supportively treated with mechanical ventilator. After assessing the ventilator parameters in the next day, a ventilator withdrawal test was performed. After removing the tracheal intubation, the patient was conscious and got out of bed without any obstacles. The oxygen flow of nasal cannula maintained at 2-3L and oxygenation remained normal. The ECMO was successfully removed on the 7th day, and the ventilator was removed after he woke up on the 8th day. On the 12th day, he was transferred to the infectious diseases department for continuous isolation and observation. Re-examination of the nucleic acid amplification tests for 2019-nCoV was negative in 2 independent tests. On the 20th day, he was considered cured and discharged.


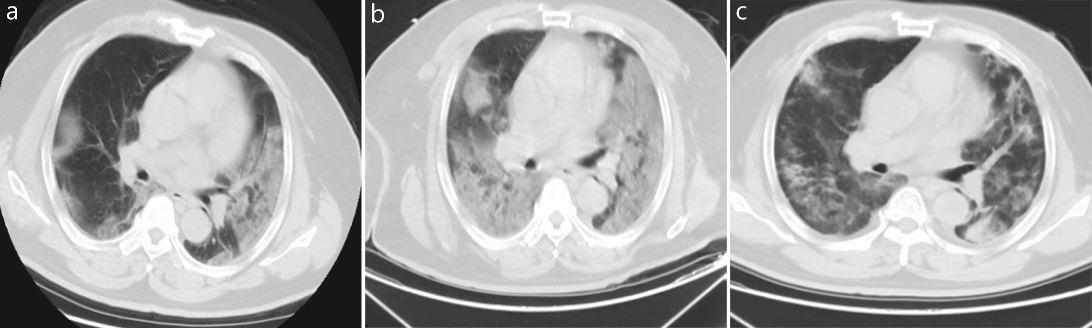


**Figure S1** Changing of CT imaging for lung

a. January 5, 2020; b. January 7, 2020; c. January 21, 2020

**
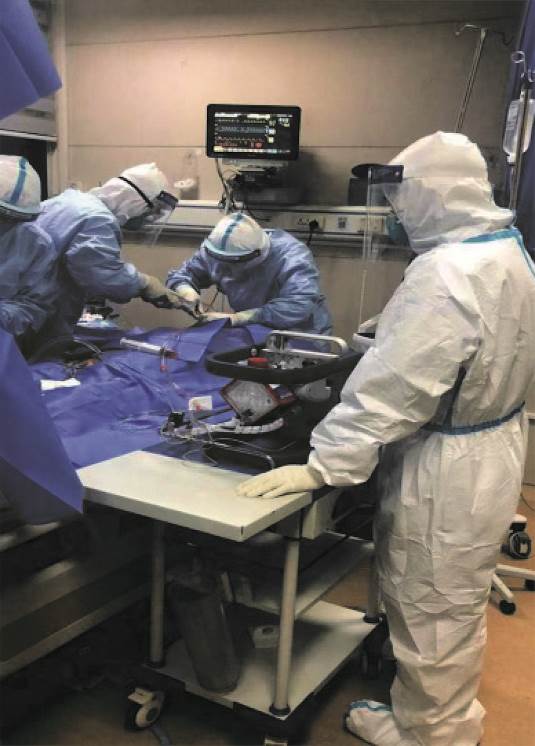
**

**Figure S2** VV-ECMO on-board process

ECMO. extracorporeal membrane oxygenation.

# 2 The successful experience of case management

## 2.1 Timely re-examination of lung imaging is an indispensable method for disease assessment

The 2019-nCoV infected pneumonia progressed rapidly, and the progress assessment by lung imaging was inconsistent with the severity of symptoms sometimes. Thus, it is necessary to re-examine the lung CT or X-ray in time (usually about 3 days) and whenever the condition changes. The patient developed dyspnea and wheezing after 3 days after admission, and re-examination of the CT image showed heavier infections, which laid the foundation of early evaluation for ECMO.

## 2.2 The early evaluation and application of ECMO is an important treatment for critical viral pneumonia

In this case, the critically illed patient with large lung lesions and severe infections might develop respiratory and circulatory failure. When general oxygen support therapy is ineffective, the early evaluation, rapid assembly, and timely operation of the ECMO team are important. Timely treatment can avoid multiple organ failure caused by hypoxia, which greatly improves the prognosis of patients. During VV-ECMO, timely adjustment of support and daily careful evaluation of the withdrawal indications are needed, striving for the early withdrawal to reduce potential complications. The extubation and mechanical ventilation withdrawal were applied after 5 days of operation, and no complications such as bleeding, bloodstream infection, and limb ischemia occurred.

## 2.3 Anticoagulation management is key to the normal operation of ECMO

Heparin was used for anticoagulation during ECMO, and the blood coagulation function was closely monitored. No complications such as systemic and ductal bleeding, or coagulation occurred.

## 2.4 Personal protection is an important guarantee for medical personnel to protect themselves

Wearing the protective equipment brings some difficulties to the medical staff when puncturing the catheter during ECMO operation. Nursing operations such as suctioning and turning over of ECMO patients will cause body fluids to splash and increase the exposure risk. The ventilator exhaust and ECMO exhaust holes especially put medical staff into high risk of exposure. Therefore personal protection is necessary to be performed to reduce medical infections. In this case, the nurse who nursed the patient was infected. That is a painful lesson.
