# Supplementary material for: A rapid advice guideline for the diagnosis and treatment of 2019 novel coronavirus (2019-nCoV) infected pneumonia (standard version)
Source: Mil Med Res. 2020 Feb 6;7:4. doi: 10.1186/s40779-020-0233-6 (PMC7003341; doi:10.1186/s40779-020-0233-6)
Supplement: Supplementary file 2 — Additional file 2. Experience and lessons in hospital rescue for 2019-nCoV infections. [file 40779_2020_233_MOESM2_ESM.doc]

# Additional file 2: Experience and lessons in hospital rescue for 2019-nCoV infections

## 1 When clustered cases are found, the emergency plan of the hospital need to be started immediately to respond quickly in the aspects of organization, information, processes, diagnosis, treatment, prevention, control, and logistics support

In this case, the capabilities to respond to the emergency and make clear the situation are necessary for large-scale public hospitals. The hospital needs to be equipped with a complete set of emergency plans, which have rapid response branches and smooth channels in the aspects of organizational structure, information relay, process management, diagnosis and treatment, infection prevention and control, and logistics support.

## 2 Standard precautionary principles, especially for workers in non-isolated areas

In this outbreak, when the source of infection and the infectivity of the virus are not yet clear, standard prevention is compulsory in the whole hospital, especially for medical staff in the non-isolation areas. It is one of the important ways to prevent medical staff from occupational exposure at the early stage.

## 3 Special attention should be paid to patients in the incubation period

In the following days of the epidemic, an outbreak of infection in the community appeared. The patient's early incubation period was longer than usual and the symptoms of infection during this period were not obvious, so it was easy to be neglected by the medical staff. Occupational exposure is extremely prone to occur if standard prevention is not conducted. So, the scope of initial screening need to be expanded and sufficient attention should be paid to the patients in the incubation period before they have a fever.

## 4 Provide timely, adequate and necessary training for the self-protection of medical staff

In the event of a public health emergency, medical staffs need to be trained urgently, including but not limited to the treatment of patients and personal protection. Training can be carried out according to the duties of medical staff: priority training should be given to key departments and high-risk departments, and then the scope of training needs to be extended to the whole hospital.

## 5 Sufficient and necessary scientific popularization for patients timely

Patients often show anxiety when the epidemic is coming. In this case, the patients should be educated with the disease-related knowledge in various ways, especially the knowledge about personal protection, home isolation and cough etiquette. Multimedia in the waiting area, information release on personal WeChat and things like that can be used as the teaching tools.

## 6 The logistics department shall ensure the smooth passage of materials

After the epidemic outbreak, the departments responsible for the logistics material security should start the pre-plan immediately, assess the material demand according to the situation, and prepare as enough materials as possible in advance. In the process of materials supply, it is necessary to optimize the practice in time and establish a working group to ensure sufficient material supplies to where needed.

## 7 Routine treatment: oxygen therapy, antiviral drugs, antibacterial drugs, glucocorticoids and symptomatic treatment

**7.1 Oxygen therapy**

The mild patients should be set up a nasal catheter to inhale oxygen. For the severe patients, a high-flow nasal catheter used for oxygen, starting at 20L / min and oxygen concentration starting from 40%, and adjust them according to oxygenation; For critically ill patients, tracheal intubation and invasive ventilator should be used to assist ventilation at VCV mode, and ventilator parameters should be adjusted based on results of blood gas monitoring.

**7.2 Choice of antiviral drugs**

In the early stage of infection, patients used Oseltamivir + Andrographolide and latter Lopinavir / Ritonavir was added.

**7.3 Choice of antimicrobials**

There are three options: (1) Amoxicillin and Flucloxacillin + Moxifloxacin; (2) Ceftriaxone and Tazobactam + Moxifloxacin; or (3) Biapenem + Moxifloxacin.

If the patient has a fever resistant to resolve, and white blood cell and neutrophil ratio and procalcitonin are significantly increased by re-examination, it is suggested to replace moxifloxacin with linezolid or vancomycin. Fungal infection should be monitored if antibiotics have been used for a long time, using antifungal drugs when necessary.

**7.4 Glucocorticoids**

For severe patients, early use of glucocorticoids with 40-80mg methylprednisolone according to the severity of the disease and body weight may help. The dosage is usually reduced after 3 days, and the course of treatment is usually 5 days. Depending on the condition and imaging performance of the patients, the treatment course can be extended as appropriate.

## 8 When the disease progresses rapidly, close observation, rechecking indicators, and adjustment of the treatment plan should be performed

The patient's vital signs (especially respiratory frequency and blood oxygen saturation) need to be closely monitored. Under normal circumstances, pulmonary CT and blood routine, electrolytes, procalcitonin and other relevant laboratory indicators need to be re-examination after 3 days to assess patient's condition and adjust the treatment plan timely. When the patient's condition changes, his family needs to be informed in a timely manner.

## 9 Home isolation of patients suspected of being infected

Facing the epidemic outbreak, the hospitals are running out their medical resource and there are too many patients waiting to be screened, it is recommended that mild to moderate suspected patients be cared and isolated at home instead of waiting in crowd in the hospital which increases the risk of infection. In the sharply increasing of patients, young and mild patients without obvious underlying diseases can also take home isolation and medication prescribed by their doctors. The prognosis is relatively good after conventional drug treatment and adequate rest.
